# Expansion of the molecular and morphological diversity of Acanthamoebidae (Centramoebida, Amoebozoa) and identification of a novel life cycle type within the group

**DOI:** 10.1186/s13062-016-0171-0

**Published:** 2016-12-28

**Authors:** Alexander K. Tice, Lora L. Shadwick, Anna Maria Fiore-Donno, Stefan Geisen, Seungho Kang, Gabriel A. Schuler, Frederick W. Spiegel, Katherine A. Wilkinson, Michael Bonkowski, Kenneth Dumack, Daniel J. G. Lahr, Eckhard Voelcker, Steffen Clauß, Junling Zhang, Matthew W. Brown

**Affiliations:** 1 Department of Biological Sciences, Mississippi State University, PO BOX GY, Mississippi State, MS 39762 USA; 2 Institute for Genomics, Biocomputing and Biotechnology, Mississippi State University, Mississippi State, MS USA; 3 Department of Biological Sciences, University of Arkansas, Fayetteville, AR USA; 4 Institute of Zoology, Department of Terrestrial Ecology, University of Cologne, Cologne, Germany; 5 Department of Terrestrial Ecology, Netherlands Institute for Ecology, Wageningen, The Netherlands; 6 Department of Zoology, Institute of Biosciences, University of São Paulo, São Paulo, 05508-090 Brazil; 7 Penard Labs, Berlin, Germany; 8 College of Resources and Environmental Sciences, China Agricultural University, 100193 Beijing, People’s Republic of China

**Keywords:** *Acanthamoeba*, Protosteloid amoebae, Amoebozoa, Protist, Protostelids

## Abstract

**Background:**

Acanthamoebidae is a “family” level amoebozoan group composed of the genera *Acanthamoeba*, *Protacanthamoeba*, and very recently *Luapeleamoeba*. This clade of amoebozoans has received considerable attention from the broader scientific community as *Acanthamoeba* spp. represent both model organisms and human pathogens. While the classical composition of the group (*Acanthamoeba* + *Protacanthamoeba*) has been well accepted due to the morphological and ultrastructural similarities of its members, the Acanthamoebidae has never been highly statistically supported in single gene phylogenetic reconstructions of Amoebozoa either by maximum likelihood (ML) or Bayesian analyses.

**Results:**

Here we show using a phylogenomic approach that the Acanthamoebidae is a fully supported monophyletic group within Amoebozoa with both ML and Bayesian analyses. We also expand the known range of morphological and life cycle diversity found in the Acanthamoebidae by demonstrating that the amoebozoans “*Protostelium*” *arachisporum*, *Dracoamoeba jormungandri* n. g. n. sp., and *Vacuolamoeba acanthoformis* n.g. n.sp., belong within the group. We also found that “*Protostelium*” *pyriformis* is clearly a species of *Acanthamoeba* making it the first reported sporocarpic member of the genus, that is, an amoeba that individually forms a walled, dormant propagule elevated by a non-cellular stalk. Our phylogenetic analyses recover a fully supported Acanthamoebidae composed of five genera. Two of these genera (*Acanthamoeba* and *Luapeleameoba*) have members that are sporocarpic.

**Conclusions:**

Our results provide high statistical support for an Acanthamoebidae that is composed of five distinct genera. This study increases the known morphological diversity of this group and shows that species of *Acanthamoeba* can include spore-bearing stages. This further illustrates the widespread nature of spore-bearing stages across the tree of Amoebozoa.

**Reviewers:**

This article was reviewed by Drs. Eugene Koonin, Purificacion Lopez-Garcia and Sandra Baldauf. Sandra Baldauf was nominated by Purificacion Lopez-Garcia, an Editorial Board member.

**Electronic supplementary material:**

The online version of this article (doi:10.1186/s13062-016-0171-0) contains supplementary material, which is available to authorized users.

## Background

Acanthamoebidae is a clade of free-living amoebae found within the Amoebozoan “order” Centramoebida (*Acanthamoeba* + *Protacanthamoeba + Balamuthia + Endostelium + Gocevia + Pellita*) [1, 2]. The Acanthamoebidae has been the focus of more scientific studies than many other amoebozoan groups owing to the medical (as caustive agent of amoebic keratitis in humans) and ecological importance (in nutrient cycling in soils) as well as the role of *A. castellanii* as a model organism [3–8]. Classically, Acanthamoebidae comprised two genera, *Acanthamoeba* and *Protacanthamoeba* [9]*.* Species of both these genera typically have flattened trophic cells that display pointed subpseudopodia (termed acanthopodia, see [10]) and a prominent lamellate microtubular organizing center (MTOC) [1, 9, 11]. Amoeboid trophic phases of *Acanthamoeba* spp. and *Protacanthamoeba* spp. have been described as nearly indistinguishable with light and electron microscopy [9, 12]. The primary character that has been used to justify the separation of the two genera has been cyst (i.e., a sessile walled dormant state) morphology [9]. However, very recently a new amoeba genus represented only by the type species, *Luapeleamoeba hula,* was incorporated into the Acanthamoebidae primarily based on the sequence of its small subunit ribosomal RNA gene (SSU) [13, 14].

*Luapeleamoeba hula* differs from *Acanthamoeba* spp. and *Protacanthamoeba* spp. not only in its general morphology (*L. hula* lacks both pointed subpseudopodia and a profile as flat as species of the aformentioned genera), but also in its life cycle complexity [14]. While in *Acanthamoeba* spp. and *Protacanthamoeba* spp. only trophic amoeboid states dividing by mitosis or encysting have been observed [9, 15], the life cycle of *L. hula* also includes the potential for individual cells to facultatively form a thin walled dormant propagule on top of a non-cellular stalk [13, 14]. The walled cellular component of this structure is known as a spore while the enitire structure (spore + non-cellular stalk) is called a sporocarp [16].

Despite organisms with amoeboid stages being present in almost all of the “kingdom” level eukaryotic assemblages, amoebae with life cycles that include the ability to form a sporocarp have so far been observed only in Amoebozoa [1, 13, 17]. Although walled propagules elevated on both cellular and non-cellular stalks are found in the amoebozoan copromyxids and dictyostelids, these structures (similarily termed sorocarps) differ in that they are the products of aggregation of many individual cells [16]. Within Amoebozoa amoebae that form sporocarps are found in a non-monohyletic group colloquially called "protosteloid amoebae" (including *L. hula*) and the monophyletic myxogastrid slime molds [1, 13, 17]. *Luapeleamoeba hula* is the most divergent organism with respect to the classical definition of acanthamoebid morphology and life history to branch within the group in molecular phylogenies. However, other amoebae with the ability to form sporocarps and/or morphologies that differ substantially from the morphology of *Acanthamoeba* spp. and *Protacanthamoeba* spp. were suggested to be allied with this amoebozoan lineage [1, 12, 18, 19].

The aim of this study was to understand better the diversity and evolutionary history of this important group of amoebozoans through molecular phylogenetic techniques and classical light microscopy. To do this we generated transcriptomes and/or SSU gene sequence data for well established (*Protacanthamoeba bohemica* [20]) and suspected acanthamoebid taxa (*Protostelium pyriformis* [21], *Protostelium arachisporum* [21]) along with two closely related centramoebids (*Pellita catalonica* ATCC® PRA25™ [22] and *Endostelium zonatum* ATCC® PRA191™ [23]) to serve as close outgroup taxa. We combined these data with previously publically available acanthamoebid data in order to clarify the phylogenetic position of the *incertae sedis* amoebozoans previously allied with Acanthamoebidae based on morphological and ultrastructural evidence [1, 18, 19], i.e., "*Protostelium" pyriformis* and *"Protostelium" arachisporum* which are here transferred to *Acanthamoeba* and *Luapelamoeba*, respectively. We also describe a new genus of acanthamoebids isolated from high altitude soils in Tibet. Finally, we provide a much needed microscopical and phylogenetic reinvestigation of ATCC® 50982™ questionably deposited as "*Stereomyxa ramosa"* [24] (here transferred to *Dracoamoeba jormungandri* n.g. n.sp.) a species also previously suggested to be a relative of acanthamoebids [12]. Our combined morphological and phylogenetic studies show the Acanthamoebidae is a highly supported lineage within the Centramoebida clade of Amoebozoa and, is composed of amoebae with a broad range of morphologies. Moreover, it includes more species with life cycles that include the ability to form sporocarps than previously known. The addition of these new taxa and the structure of our trees suggest that the simplistic classical acanthamoebid life cycle could potentially be derived from an ancestor with a more complex life cycle. This evolutionary trend of derived simplicity both morphologically and genomically is not only seen in Amoebozoa, but scattered across the Tree of Life as a whole [25, 26].

## Methods

### Strains examined

Applicable information for all strains examined in this study including: former and newly proposed taxonomic assignment, culture collection information, isolator, isolation habitat and location, and the type of data generated in this study can be found in Table 1. Details on culture maintenance can be found in materials and methods section in Additional file 1.

**Table 1:** Taxonomic, isolation, and data generation information for all isolates used in this study

Species Examined	Proposed New Name	Strain	Culture Collection	Accession Number	Isolator/Depositor	Habitat	Collection Location	Coordinates	Data Collected
*Protostelium pyriformis*	*Acanthamoeba pyriformis* n. comb.	CR15	CCAP	1501/19	K.Wilkinson/M.W. Brown	Leaf litter	Costa Rica	8.783333° N -82.966667° E	morphological, SSU, RNA
*Protostelium arachisporum*	*Luapeleamoeba arachisporum* n. comb.	OG15	CCAP	2545/1	M.W. Brown/M.W. Brown	Leaf litter	Winston County, Mississippii (USA)	33.219118° N -89.098003° E	morphological, SSU, RNA
*Protostelium arachisporum*	*Luapeleamoeba arachisporum* n. comb.	CR15	NA	NA	A.K. Tice/NA	Leaf litter	Costa Rica	8.783333° N -82.966667° E	morphological, SSU
*Protostelium arachisporum*	*Luapeleamoeba arachisporum* n. comb.	AMFD	NA	NA	A.M. Fiore-Donno/NA	Leaf litter	Geneva, Switzerland	46.1358° N 5.9695° E	morphological, SSU
*Protostelium arachisporum*	*Luapeleamoeba arachisporum* n. comb.	PKB06-4 L-1	NA	NA	M.W. Brown/NA	Leaf litter	Pilot's Knob, Arkansas (USA)	36.240990° N -93.225232° E	morphological, SSU
Unknown soil amoeba	*Vacuolamoeba acanthoformis* n.g. n.sp.	Tib 84	CCAP	2580/1	K. Dumack/M.W. Brown	High Altitude Soil	Tibet	29.700000° N 92.166667° E	morphological, SSU
Unknown soil amoeba	*Vacuolamoeba* sp. n.g. .	Tib 168	NA	NA	K. Dumack/NA	High Altitude Soil	Tibet	29.866667° N 92.550000° E	SSU
*Stereomyxa ramosa*	*Dracoamoeba jomungandri* n.g. n.sp.	Chinc5	ATCC	50982	T.A. Nerad/T.A. Nerad	moist soil from mud flat approximately 800 yards from the ocean	Chincoteague, Virginia (USA)	NA	morphological, SSU (bioinformatically)
*Luapeleamoeba hula*	Not Applicable	LHI05	ATCC	PRA-198	L.L. Shadwick/F.W. Spiegel	Leaf litter	Hawaii (USA)	NA	morphological, RNA
*Protacanthamoeba bohemica*	Not Applicable	TT3H	Institue of Parasitology, Academy of Sciences of the Czech Republic, Ceske Budejovice	NA	I. Dykova	Liver of *Tinca Tinca*	Spolský pond, South Bohemia (Czech Republic)	NA	morphological, RNA
*Endostelium zonatum*	Not Applicable	LHI05M6a-1	ATCC	PRA-191	L.L. Shadwick/F.W. Spiegel	Leaf litter	Hawaii (USA)	NA	RNA
*Pellita catalonica*	Not Applicable	CON-1	ATCC	PRA-25	T.A. Nerad/T.A. Nerad	carapace of an American lobster, *Homarus americanus*	Conneticut (USA)	NA	RNA

### Light microscopy

All life cycle stages of all organisms were imaged with a Zeiss Axioskop2 Plus or an AxioVert 135 (Zeiss, Peabody, MA) equipped with 10X and 40X lenses capable of DIC and 10X and 32X lenses capable of phase contrast, respectively. Digital photographs were taken using either a Canon EOS 650D or Canon 5DS (Canon, Melville, NY ).

### cDNA library preparation and next-generation sequencing

Total RNA was extracted and ds-cDNA constructed using a modified version of Smart-seq2 [27] for *A. pyriformis* isolate CR15 and *P. bohemica* isolate TT3H. For *L. arachisporum* OG15, *L. hula* ATCC® PRA198™, *E. zonatum* ATCC® PRA191™, and *P. catalonica* ATCC® PRA25™ Poly(A) + RNA was isolated through Poly(A) + selection. A paired-end cDNA library with a nominal insert size of ~375 bp was then constructed with NEBNext Ultra RNA Library Prep Kit for Illumina (New England Biolabs). Modifications made to Smart-seq2 and quality assessment steps involving RNA and cDNA can be found in supplementary Materials and Methods in Additional file 1. Libraries were diluted and manually pooled with other uniquely indexed libraries according to Illumina specifications. The library pools were sequenced on either HiSeq 2000 or HiSeq 2500 at Genome Quebec.

### Transcriptome assembly

Raw sequence read data were filtered based on quality scores with the Trimmomatic program [28], using a cutoff filter (a minimum 70% of bases must have quality of 20 or greater). Filtered sequences were assembled into clusters using TRINITY 2.1.1 package [29] as per standard developer’s protocols.

### Acquisition of SSU rDNA sequences

For *A. pyriformis* isolate CR15, *L. arachisporum* isolates PKB06-4 L-1, AMFD and CR15 total genomic DNA was extracted from established clonal cultures and the SSU gene was amplified through polymerase chain reation and sequenced by Sanger sequencing. The partial SSU sequences of *Vaculoamoeba acanthaformis* Tib84 and *Vacuolamoeba* sp. Tib168 were acquired as in [5]. The SSU sequences of *Dracoamoeba jormungandri* n.g. n.sp. and *L. arachisporum* isolate OG15 were acquired bioinformatically from their respective transcriptomes. Detailed decriptions of DNA extraction methods, primers used for PCR, thermocycler conditions, and bioinformatic stratagy for obtaining SSU sequences from transcriptomes can be found in supplementary materials and methods in Additional file 1.

### Phylogenetic analyses

#### Phylogenomic matrix construction

The transcriptomic data, as mentioned above, were used as inputs for an in-house pipeline, described below, for the creation of single protein datasets and, subsequently, the phylogenomic data matrix. The organismal data were individually screened for orthologs using either blastp (1e-5 e-value cutoff) with a manually curated reference of 325 ortholog sequences as queries in BLASTMONKEY from the Barrel-o-Monkeys toolkit [30] (Additional file 2). Blastp was then used to screen these putative orthologs against the OrthoMCL database, and the output for each gene from each organism was compared against a manually curated dictionary of orthologous OrthoMCL IDs. Those putative orthologs that did not match orthologous IDs were designated as paralogs and removed. The remaining putative orthologs from each organism were combined and aligned using MAFFT-LINSI [31]. Ambiguously aligned positions were identified and removed using Block Mapping and Gathering with Entropy (BMGE) [32] (unmasked alignment files, masked alignment files, the supermatrix, and single gene trees are available in Additional file 3). For each individual protein alignment, maximum-likelihood (ML) trees were inferred in RAxML v8 [33] using an LG model [34] with four categories of among-site rate variation, with 10 ML tree searches and 100 ML bootstrap replicates. To test for undetected paralogy or contaminants, we constructed a consensus tree (ConTree) representing phylogenetic groupings of well-established eukaryotic clades [35]. The resulting individual protein trees that placed taxa in conflicting positions relative to the ConTree with more than 70% ML bootstrap support, with a zero-branch length, or with extremely long branches were checked manually. All problematic sequences identified using these methods were removed from the dataset. The resulting protein alignments were then re-trimmed for ambiguously aligned positions using BMGE and concatenated into the separate supermatrix with 102,140 amino acid sites (325 proteins) of 40 taxa using alvert.py from the Barrel-o-Monkey’s toolkit.

Bayesian phylogenetic analyses were performed using Phylobayes-MPI v1.6j [36, 37] under the site heterogeneous exchangeability CAT-GTR model of protein evolution on the phylogenomic matrix. Six independent Markov chain Monte Carlo chains were run in Phylobayes-MPI for ~2700 generations, sampling trees every two generations. After 1200 generations convergence was achieved for two of the six chains. These two chains were summarized with the largest discrepancy in posterior probabilities (PPs) (maxdiff) less than 0.012 and the effective size of continuous model parameters were in the range of acceptable values. The other four chains that did not converge with a maxdiff of 1.0 do not differ in the placement of our taxa of interest and are summarized in Additional file 4 after a 1200 generation burnin. In additon to the Bayesian analyses, we employed C-series models [34] that account for heterogeneous site-specific features of sequence evolution in the phylogenomic dataset under a maximum-likelihood (ML) framework in IQ-TREE v1.3.3 [38]. The best-fitting model available under ML analyses that we were capable of running with computational constraints was LG + Γ4 + C20 + F with class weights optimized from the dataset using the exchangeabilities from the LG Q-Matrix (LG + Γ4 + FMIX (empirical, C20pi1-C20pi10)) [39, 40]. Topological support was estimated from 1,000 ultrafast ML bootstrap (ML BS) replicates in IQ-TREE.

#### Fast evolving site removal

Our phylogenomic dataset composed of 325 genes from 40 taxa resulted in a 102,140 amino acid (AA) site concatenated supermatrix (Additional file 4). We also evaluated the impact of removing the fastest evolving sites from the supermatrix, which are expected to be the most prone to systematic error in phylogenomic analyses [35]. To do this, rates of evolution per site were estimated with Dist_Est [41] under the LG + Γ4 model using discrete gamma probability estimation. Then a custom Python pipeline [35] was used to remove fastest evolving sites in a stepwise fashion (3,300 sites per step). Each step was analyzed using 100 MLBS pseudoreplicates in IQ-Tree under LG + Γ4 + F which are plotted in Additional file 5.

#### SSU rDNA phylogenetics

Small subunit rRNA genes were aligned using MAFFT [31],and ambiguous sites were removed by hand in Seaview [42]. Maximum liklihood phylogenies of the SSU gene were built using RAxML v8.2.4 [33] and Bayesian analyses were carried out by MrBayes v3.2.6 [43]. In both instances a GTR + Γ + I model of nucleotide substitution was used. Further detials can be found in the supplementary materials and methods section of Additional file 1.

## Results

### Light microscopy

#### *Acanthamoeba pyriformis* n. comb. & *Luapeleamoeba arachisporum* n. comb.

**Fig. 1:**
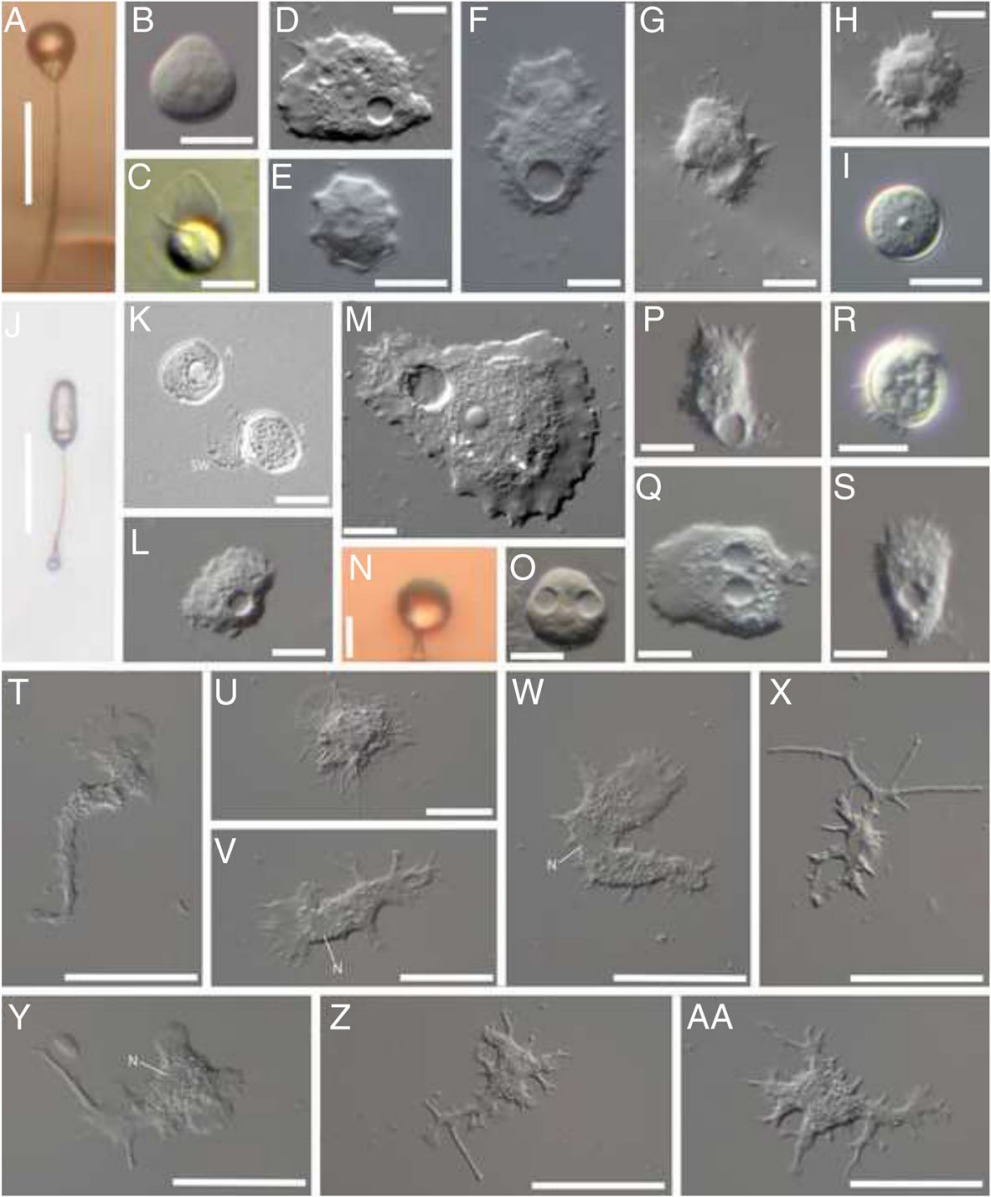
Acanthamoebids observed in this study. **a-f**. *Acanthamoeba pyriformis* n. comb. CR15: **a**) Sporocarp; **b**) Spore detached from stalk; **c**) Empty spore wall and rounded trophic cell; **d**) Trophic cell; **e**) Cyst; **f**) Trophic cell; **g-i**. *Protacanthamoeba bohemica* TT3H: **g**) Trophic cell; **h**) Trophic cell; **i**) Cyst; **j-l**. *Luapeleamoeba arachisporum* n. comb. CR15: **j**) Sporocarp; **k**) Amoeba moments after germination, empty spore wall, and ungerminated spore; **l**) Trophic cell; **m-o**. *Luapeleamoeba hula* ATCC® PRA198™: **m**) Trophic cell; **n**) Sporocarp; **o**) Spore detached from stalk; **p-s**. *Vacuolamoeba acanthoformis* n.g. n. sp. Tib 84: **p-q & s**) Trophic cell; **r**) Cyst; **T-AA**. *Dracoamoeba jomungandri* n. g. n. sp. ATCC® 50982™ **T-AA**) Trophic cell. A = amoeba, S = spore, SW = spore wall, N = nucleus

**Fig. 2:**
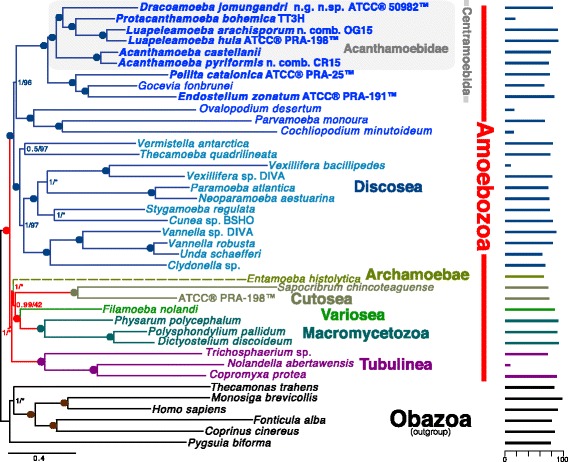
325 gene (102,140 AA sites) phylogeny of Amoebozoa rooted with Obazoa. The tree was built using PhyloBayes-MPI v1.5a under the CAT + GTR model of protein evolution. Values at nodes are posterior probability and ML bootstrap (BS) (1000 ultrafast BS reps, IQ-Tree LG + Γ4 + FMIX(emprical,C20)) values respectively. Circles at nodes represents full support in both analyses (1.0/100). Nodes not recovered in the corresponding ML analysis are represented by an asterisk. The length of the *Entamoeba* branch is shown as a dashed line to represent that its total length has been reduced by 50%. Bars along the right side of the figure show the percent of the total data set available for each taxon. Novel data was generated in this study for taxa whose names are bold

**Fig. 3:**
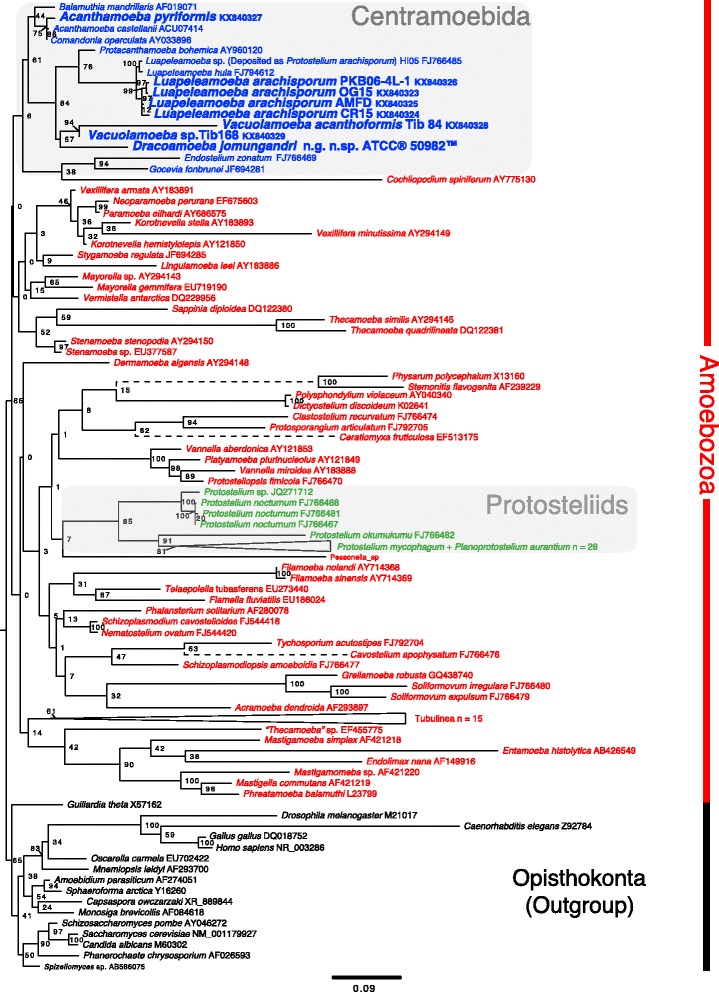
Maximum likelihood phylogeny of Amoebozoa rooted with Ophisthokonta based on the SSU gene and 1,326 nucleotide positions. The tree was constructed under a GTR + Γ + I model of nucleotide substitution. The Centramoebida and Protosteliid clades are highlighted and taxa of interest are in bold. Values at nodes are maximum likelihood bootstrap values. The length of branches depicted as dashed lines have been reduced by 50% for presentation purposes. A full version of this figure showing all sequences and their accessions numbers included in collapsed clades is shown in Additional file 6

**Fig. 4:**
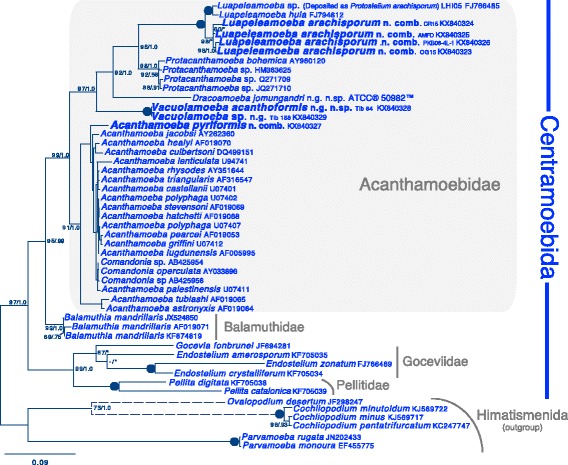
Maximum likelihood SSU phylogeny of 43 centramoebid taxa and six outgroup himatismenid taxa. A GTR + Γ + I model of nucleotide substitution was used and 1,617 unambiguously aligned sites were included. Newly sequenced taxa are in bold. ML bootstrap and Bayesian PP values are given for each node, black dots represent full support (100/1.0). Support values less than 50/.50 are represented with a dash and nodes not recovered in one of the analyses are represented by an asterisk. Internal support values for the clade of *Acanthamoeba* spp. are not shown for graphical limitation

Morphological details confirming the identity of our isolates obtained from nature of these two species (originally described as *Protostelium pyriformis* (Fig. 1: a-f) and *Protostelium arachisporum* (Fig. 1: j-l) respectively) can be found in supplementary results. See Figs. 2, 3, 4, and Additional file 6 for justification of reassignment of each to different previously diagnosed genera.

#### *Vacuolamoeba acanthaformis* n. g. n. sp.

The majority of the body of the cell consists of granuloplasm (cytoplasm with inclusions such as organelles) while the leading edges of cells in motion are made up of hyaloplasm (clear cytoplasm lacking any inclusions). Acutely pointed subpseudopodia (i.e., acanthopodia) project outwards from all sides of the cell body. Cells are typically uninucleate although binucleate individuals were sometimes observed. Cells typically have many vacuoles in the granuloplasm; among them one or more large round contractile vacuoles are usually present (Fig. 1 p, q and s). Cell motion is slow, but easily visible when observed under the microscope. Uroids (distinct arrangements of cellular extensions at the posterior end of some amoeba species) have rarely been observed. Cells readily form cysts in culture. These cysts are round to slightly irregular in shape and consist of what appears at the light microscope level to be a single smooth wall enclosing granuloplasm (Fig. 1r). The cysts were most often seen to form singly rather than in clusters.

#### *Dracoamoeba jormungandri* n. g. n. sp. ATCC® 50982™ (deposited as *"Stereomyxa ramosa"*)

The amoebae of ATCC® 50982™ are highly variable in their morphology (Fig. 1t-aa) and most of the time do not resemble classical acanthamoebids. On occasion subpseudopodia that resemble acanthopodia form (Fig. 1v and w). The cell body is composed mainly of granuloplasm while pseudopods are clear (hyaloplasmic). Only uninucleate individuals were seen. When observed, the nucleus appears as an irregular clear spot in the cell (Fig. 1 v, w and y). No nucleoli are obvious using light microscopy. As expected for a marine organism, amoebae were never seen to form contractile vacuoles. No cysts or resting stages were ever seen in our cultures. Amoebae move extremely slowly and can be observed using time-lapse microscopy. Uroids were never observed. No anastomosis or any form of fusion of pseudopodia within or between individuals was ever observed. The characteristics of this organism do not adequately fit the original description given by [24] for *Stereomyxa ramosa* and so we establish it here as the new genus *Dracoamoeba* n. g. and designate this isolate the type species *Dracoamoeba jomungandri* n. g. n. sp. For a full discussion on the inconsistencies between our observations and those of Grell see the supplemental discussion (Addtional file 1).

Additional light microscope observations on *Vacuolamoeba acanthaformis* and *Dracoamoeba jomungandri* can be found in the results section of Additional file 1.

### Phylogenetic analyses

#### 325 gene analyses

The results of Bayesian analysis using 325 protein-coding genes (102,140 amino acid sites) from 34 amoebozoan taxa and 6 obazoan taxa as outgroup are presented in Fig. 2. We recover a fully supported (1.0 Bayesian posterior probability/100 ML bootstrap) Acanthamoebidae lineage of Centramoebida that includes our taxa of interest. Despite the morphologcial similarities shared by the amoebae of *Protacanthamoeba* spp. and *Acanthamoeba* spp. we do not recover a sister relationship between the two representative species in our analyses. Instead we show the deepest bifurcation lies between *Acanthamoeba* spp. and all other taxa. Similar to the analyses of [13] *Luapeleamoeba* spp. are sister to *Protacanthamoeba bohemica*. This clade (*Luapeleamoeba* + *Protacanthamoeba*) is sister to *Dracoamoeba jomungandri* ATCC® 50982™*.* All internal Acanthamoebidae relationships are fully supported.

#### SSU only analyses

In our analysis of the amoebozoan-wide SSU data set that includes the 34 publicly available *Protostelium* and *Planoprotostelium* (Protosteliida *sensu* Shadwick et Spiegel in [1]) sequences, only one (deposited as *Protostelium arachisporum*) had any phylogenetic affinity with our isolates of *Acanthamoeba pyriformis* and *Luapeleamoeba arachisporum* (formally *Protostelium pyriforms* and *Protostelium arachisporum* respectively) Fig. 3. All of our isolates branch within the Centramoebida with high support although the exact internal branching order of the group is not well resolved and both the Centramoebida and Acanthamoebidae are paraphyletic with low support (Fig. 3). All other *Protostelium* spp. and *Planoprotostelium aurantium* sequences form a separate highly supported monophyletic group Fig. 3. We also show in our amoebozoan-wide analysis that *Dracoamoeba jomungandri* ATCC® 50982™ and soil isolates Tib 84 and Tib 168 are also members of the Centramoebida (Fig. 3). A full version of Fig. 3 showing all taxa included in collapsed clades is shown in Additional file 6.

Analyses using the centramoebid-enriched data set shows a highly supported (97/1.0) Acanthamoebidae that is composed of five genera (*Luapeleamoeba, Protacanthamoeba*, *Dracoamoeba*, *Vacuolamoeba,* and *Acanthamoeba*) Fig. 4. As in our multigene analysis we do not recover a sister relationship between *Protacanthamoeba* and *Acanthamoeba*, but instead show with high support the deepest bifurcation in the Acanthamoebidae lies between *Acanthamoeba* and the other four genera. Our isolates of *L. arachisporum* that precisely fit the description of the type strain [16, 44] form a monophyletic group while a sequence deposited on GenBank as *Protostelium arachipsorum* (labelled in our trees as *Luapeleamoeba* sp.) branches outside the group sister to *L. hula* with full support. *Vacuolamoeba* spp. constitute a novel distinct lineage within the Acanthamoebidae.

## Discussion

### Acanthamoebid phylogeny and classification

We show conclusively through molecular phylogenetic analyses that *Luapeleamoeba arachisporum* n. comb*.*, *Vacuolamoeba* n. g. spp., *Dracoamoeba jomungandri* n. g. n. sp. ATCC® 50982™, and *Acanthamoeba pyriformis* n. comb. belong to a clade that includes the classical Acanthamoebidae (*Acanthamoeba* and *Protacanthamoeba*). The addition of these taxa demonstrates that the group is more diverse than previously known with respect to the morphology of its amoebae and the life cycles observed in its members as well as the environments from which they can be isolated. Thus, here we revise the concept of Acanthamoebidae [11].

In the orignal circumscription of Acanthamoebidae [11], the group was based solely on morphological and ultrastructural characteristics uniting *Acanthamoeba* spp. The amoeba of these taxa are somewhat flattened amoebae with pointed subpsuedopodia (acanthopodia) produced from broad rounded psuedopodia. All members also make cysts that are multilayered. Page [9] enlarged the family to include the genus *Protacanthamoeba*. The major distintion between the genera is cyst morphology [9]. These are irregular and operculate in *Acanthamoeba* and round and inoperculate in *Protoacanthamoeba*. Ultrastructually, both genera have a distinct, laminate MTOC in the form of a plaque- or bar-shaped body [9, 11, 20]. Recently, Shadwick et al. [14] added the genus *Luapeleamoeba,* with its single described species, *L. hula,* to the family Acanthamoebidae. They based this primarily on molecular analyses [13, 14], given that the amoebae do not form acanthopodia and there are essentially no cysts in *L. hula*. However, there is a simplified interphase MTOC [14]. In Shadwick et al. [13, 14], as well as here, Acanthamoebidae are paraphyletic with respect to *Acanthamoeba* and *Protacanthamoeba* with the exclusion of *L. hula* which is shown to be sister to *P. bohemica.* We propose thus to expand Acanthamoebidae to include these morphologically and molecularly diverse taxa, providing a diagnosis at the end of the text.

Two of the primary taxa examined here and placed in Acanthamoebidae (*Acanthamoeba pyriformis* and *Luapeleamoeba arachisporum*) were previously classified in the sporocarpic genus *Protostelium*. The majority of protosteliod amoeba species were described by Olive [15] uniquely on their faculty to build sporocarps, regardless of the amoebal morphology. The sporocarp characteristics and in a lesser extent some ameobal characteristics were used to define genera. *Protostelium mycophaga* was the type of the genus [16, 21, 44, 45], whose characteristics were a relatively long-stalked sprorocarp with a deciduous spore, arising from an uninucleate amoeba (detailed in the depth in [16]). We provide new taxonomic homes for these two species (*"P." arachisporum* and *"P." pyriformis)* here in *Luapeleamoeba* and *Acanthamoeba*, respectively.

**Fig. 5:**
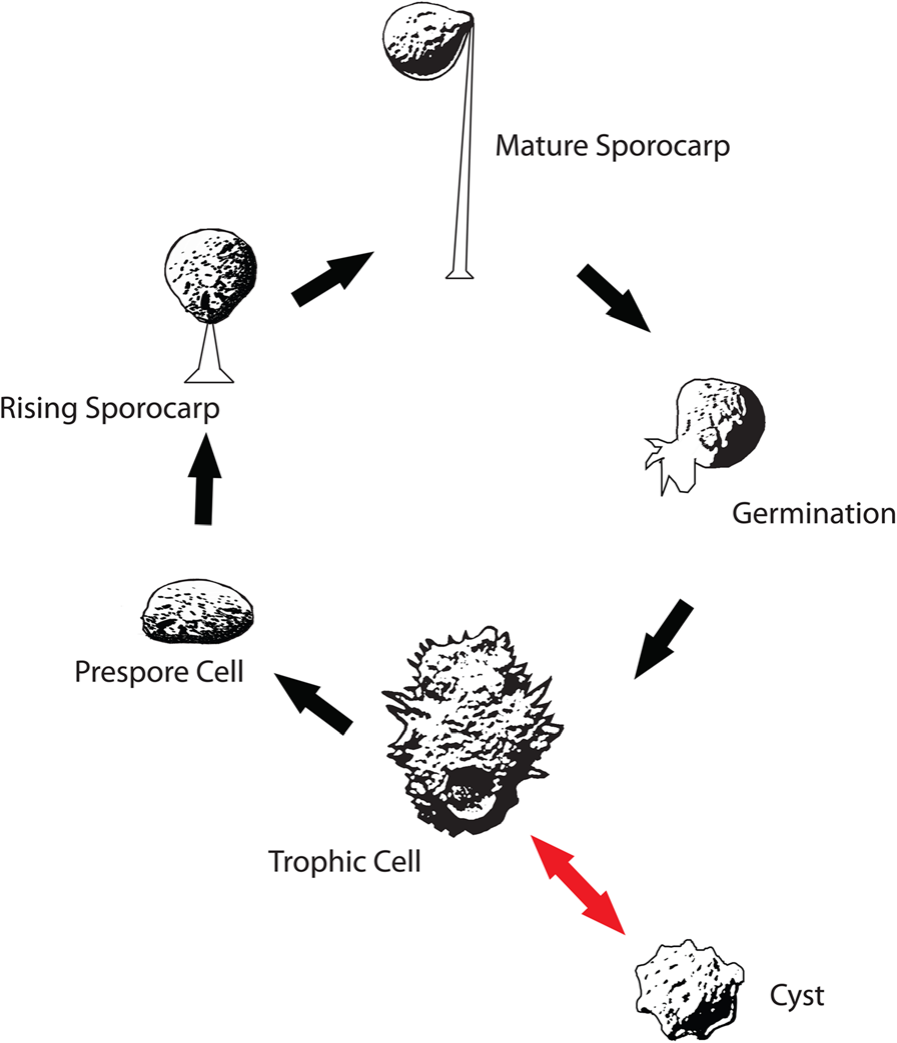
Diagrammatic representation of the life cycle of *Acanthamoeba pyriformis*. Red arrows indicate the known life cycle of all other *Acanthamoeba* spp.

*Acanthamoeba pyriformis* is the first species of *Acanthamoeba* recognized to include facultative sporocarpic fruiting in its life cycle (Fig. 5). Prior to this study, the life cycle of all *Acanthamoeba* spp. (see [46] for variation within the genus) was limited to an active trophic amoeba, dividing following mitosis and forming a complex cyst in adverse conditions (Fig. 4). Whether sporocarpy exists but has gone unobserved in other *Acanthamoeba* spp. cannot yet be decided. Hypothetically, sporocarpy could exist in more species, since most isolation methods for *Acanthamoeba* use liquid media, where sporocarps cannot develop. Also, sporocarps could be taken for a contamination or remain unnoticed. Searches for new protosteloid amoebae, especially ones that resemble variations on the theme of *A. pyriformis*, using the standard methods of collection for protosteloid amoebae [16, 47–49] may be the most fruitful way to address this point because we often observe *Acanthamoeba* spp. in these collections. In addition, maintenance of more amoeba cultures on low nutrient agar media may stimulate fruiting in not only more *Acanthamoeba* spp. but also other amoebozoan lineages as well. The introduction of substrates such as sterilized plant tissues (bark and/or leaves) or pollen into agar cultures of amoebae has been shown to induce both sporocarpic and sorocarpic fruiting (aggregation of many cells that leads to the production of a subaerial spore-bearing stalked structure), in amoebae known to fruit and in others not previously known to do so [23, 50]. However, even on low nutrient agar media we have noticed that many cultures of phylogenetically diverse sporocarpic amoebae periodically “lose” the ability to produce sporocarps only to “regain” it days, months, or even years later (personal observations of FWS, AKT, LLS, and MWB). This “loss” and “gain” of sporocarps is likely due to fluctuations in the microenvironment (especially with regards to humidity and/or the buildup of volatile compounds in cultures when the plates are sealed with Parafilm). Until the SSU gene sequences of more isolates resembling *A. pyriformis* are available, it is not possible to know if many different *Acanthamoeba* spp. have been incorrectly identified as *A. pyriformis* based on the production of a sporocarp, or if sporocarp production in *Acanthamoeba* is truly unique to this species alone. It is worth noting that most nonclinical *Acanthamoeba* spp. isolates are from either soil or aquatic environments. *Acanthamoeba pyriformis*, like nearly all protosteloid amoebae, is globally distributed on decaying plant leaves and/or tree bark [13, 48, 51–54]. These environments, to our knowledge, have not been surveyed for the presence of *Acanthamoeba* spp. and may harbor additional species (see [47, 48] and [49] for methods).

Currently, all recognized species of *Luapeleamoeba* display sporocarpic fruiting [14] and it was *L. hula*, as undescribed species LHI05, that was referred to as a known sporocarpic member of Centramoebida in [1]. Amoebae of *L. arachisporum* are much more similar to those of *L. hula* than they are to those of *Protacanthamoeba* (Fig. 1g-o).

Despite the slower motilty and the morphological variation of *Dracoamoeba jomungandri* compared to other acanthamoebids, *Dracoamoeba* does display acanthopodia at times (Fig. 1: t, v, w). Through careful examination of the transcriptomic data and culture observations, there is no reason to believe that contamination is responsible for the phylogenetic attraction of this organism to the acanthamoebids. Additional work to examine the fine structure of *Dracoamoeba* and *Vacuolamoeba* should be pursued to search for the presence of interphase MTOC’s, like those found in *Luapeleamoeba* [14], *Acanthamoeba* (i.e., [46]), and *Protacanthamoeba* (i.e., [46]). However, given the structure of both our single and multigene phylogenetic trees, knowledge of the presence or absence of an interphase MTOC is not necessary to include the taxa in the acanthamoebid lineage of Centramoebida.

Presently, formal descriptions of both *Luapeleamoeba* sp. (deposited on GenBank as "*P. arachisporum"* under FJ766485) and *Vacuolamoeba* sp. (Tib 168) are here foregone, each for a different reason. A culture of *Luapeleamoeba* sp. (FJ766485) is no longer available. Although high quality micrographs of the amoebae of this species exist, unfortunately only poor qualtiy low magnification micrographs of sporocarps are available [55]. However, the sporocarps of this species have only ovate spores rather than peanut-shaped spores. Since sporocarp characteristics are of primary importance to identify protosteloid amoebae, additional isolations and micrographs correlated with sequences are needed for a proper description. We also do not assign *Vacuolamoeba* sp. to the newly described species *V. acanthoformis*, since only partial SSU sequences were obtained.

### Origins of Acanthamoebidae and Centramoebida

The unexpected diversity of Acanthamoebidae revelaed by the present study has interesting implications for possible evolutionary patterns. One of the most interesting of these is suggested by presence of protosteloid sporocarpic fruiting scattered among the Acanthamoebidae and their centramoebid relatives.

The ability to form sporocarps is found in phylogenetically and morphologically diverse clades across the tree of Amoebozoa [13, 56], and, so far, no where else in eukaryotes, despite concerted efforts to find them. As more phylogenetic data on more amoebozoan species accumulate it seems that sporocarpic taxa are far more widely distributed across groups of amoebae than previously suspected [13, 22, 57]. The present study now extends this to the Acanthamoebidae showing that it includes both sporocarpic amoebae and apparently non-sporocarpic taxa. By doing so we continue to show this trend also exists in the Acanthamoebidae and the Centramoebida *sensu* [2] as a whole, being found in *Acanthamoeba*, *Luapeleamoeba*, and *Endostelium*. The origin and evolution of sporocarpic fruiting is contentious among amoebozoan researchers [13, 57, 58]. This is further complicated by the patchy distribution of sporocarpic fruiting and a complete lack of information on molecular and physiological traits that induce and regulate sporocarp formation. Thus Centramoebida have a number of characteristics that make them potentially useful for testing hypotheses about the evolution of sporocarpy.

Within the Acanthamoebidae or more broadly in the Centramoebida, sporocarpic taxa share a number of common developmental features. The sporadic distribution of these features suggest the possibility that sporocarpic fruiting had a common origin in these clades. For example, in both *A. pyriformis* and *L. hula* the sporocarp stalk forms in an invagination of the developing sporocarp [14, 58]. Fruiting development in *L. arachisporum* and *L.* sp. has not been studied in enough detail to know whether this is the case in these species as well. Development in the fruiting pellitid amoebozoans (Pellitdae) in the genus *Endostelium* is similar to that seen in both *A. pyriformis* and *L. hula* [13, 14, 23, 44, 49]. All fruiting centramoebids, *sensu* [2] have stalks with a solid, knob-like apophysis that inserts into an invagination in the mature spore [13, 14, 23, 44, 49]. It must be noted that in no species of protosteloid centramoebid do spores in any way correlate with cyst morphology, e.g., compare Fig. 1 a-c to e. If these common features are homologous as current, albeit still limited evidence suggests, then the most reasonable interpretation would be that the last common ancestor of centramoebids was a protosteloid amoeba, and that those exclusively non-fruiting members would have lost this ability. Thus, centramoebids could prove to be a useful model system to understand evolution of gains and losses of complexity.

Potential losses of sporocarpic fruiting acanthamoebids is consistent with what appears to be a evolutionary trend across Amoebozoa. That is the loss of complexity in the descendants of more complex common ancestors. Unequivocal examples of such loss of complexity are already documented for Amoebozoa, e.g., multiple losses of sex [56, 59] and flagellate states [13].

## Conclusions

Our expansion of Acantamoebidae in this study increases the known diverisity in morphology, life history, and phylogenetic depth of a clinically and environmentally important group. These results illustrate that Acanthamoebidae has the potential to be model system is representative of evolutionary trends in Amoebozoa and in studies interested in genome reduction leading to loss of complexity.

## Taxonomic appendix

***Acanthamoeba pyriformis*** n. comb. (Olive & Stoianovitch 1969) Spiegel & L. Shadwick 2016

Due to the lack of type material (strain NE-65-67, Olive and Stoianovitch 1969) availability we are designating strain CR15 as a neotype specimen.

**Neotype material:** Type culture was deposited at CCAP accession number 1501/19.

**Neotype habitat:** leaf litter from a deciduous forest in Costa Rica.

**Neotype sequence:** The partial SSU of the type strain has been deposited on NCBI GenBank accession number KX840327.

**Description**: Sporocarp morphometrics were not taken for this material because all fruiting bodies fell within the known size range reported by Olive and Stoianovitch (1969) [44] for the original isolate; however, since they did not carefully describe the amoebae and cysts in their study, we here provide a more detailed description of these cells.

During locomotion amoebae are flat in cross section and vary from nearly circular in outline to flabellate to elongate to sometimes branching. Locomoting amoebae are typically longer than they are wide along the axis of motility, but may occasionally be wider than long. Mean cell length is 26.9 μm (standard deviation = 4.2 μm, *n* = 30) and mean cell breath is 19.3 μm (standard deviation = 3.8 μm, *n* = 30). The leading edge of the locomoting amoeba is a lobose, hyaloplasmic pseudopodium that typically supports acanthapodia. The pseudopodium usually makes up 20-25% of the length of the amoeba. Acanthopodia may extend from all around the circumference of the cell. There is typically no uroid. The granular cytoplasm contains a single, spherical to subspherical nucleus (mean diameter is 5.1 μm) with a central to slightly eccentric nucleolus (mean diameter is 2.3 μm) that is never more than half the diameter of the nucleus, and often less. There is usually a single contractile vacuole that is typically located posterior to the nucleus in locomoting cells within a distance of one nuclear diameter. At diastole, the contractile vacuole is equal to or greater in diameter than the nucleus. When cells round up during mitosis (not illustrated), they become circular in outline with short acanthapodia radiating from their entire circumference. These acanthapodia are present from prophase through early cytokinesis. Cysts are mostly isodiametric with stellate knobs, with a mean diameter of 13.1 μm (*n* = 3). The cyst walls appear to have only one wall layer when viewed with light microscopy. Sporocarps develop from a prespore cell that develops as an amoeba rounds up and becomes refractile. Just before stalk deposition begins the prespore cell assumes an ellipsoid shape then becomes nearly spherical. The prespore transitions to the sporogen stage as stalk deposition begins. The sporogen is obpyriform, and the stalk is deposited in an invagination of the narrowed lower portion of the sporogen. At maturity, the sporogen lays down a spore wall and becomes an obpyriform spore with an invagination into which the apex of the stalk is inserted. The spore is deciduous and is easily removed from the stalk by air currents. Before the spore is shed, it waves around, flags, readily in air currents. The stalk is several times the diameter of the spore in length and tapers upward from a distinct basal disk to a narrow column. When the spore is shed, the apex of the stalk, which was inserted into the invagination of the spore, can be seen to swell into a knob-like swelling, the apophysis, that appear to be solid when viewed with light microscopy.

***Luapeleamoeba arachisporum*** n. comb. (Olive & Stoianovitch 1969) Tice & Brown 2016

Due to the lack of type material (strain Hi-49, [44]) availability we are designating strain OG15 as a neotype specimen.

**Neotype material:** Type culture was deposited at CCAP accession number 2545/1.

**Neotype habitat:** leaf litter from a deciduous forest in Mississippi, USA.

**Neotype sequence:** The partial SSU of the type strain has been deposited on NCBI GenBank accession number KX840323.

**Description:** Sporocarp morphometrics were not taken for this material because all fruiting bodies fell within the known size range reported by Olive and Stoianovitch (1969) [44] for the original isolate; however, since they did not carefully describe the amoebae and cysts in their study, we here provide a more detailed description of these cells.

During locomotion, the amoebae are shallowly dome-shaped in cross section, resembling a shield volcano (thus the genus name Shadwick et al. 2016). Locomoting amoebae range from nearly circular in outline to flabellate to elongate. Amoebae are as often wider than long as longer than wide with respect to the axis of locomotion. Mean cell length is 19.8 μm (standard deviation = 2.58 μm, *n* = 33) and mean cell breath is 15.4 μm (standard deviation = 2.78 μm, *n* = 33). The leading edge of a locomoting amoeba consists of a broad, hyaloplasmic, lobose pseudopodium from which extend numerous short, triangular, blunt subpseudopodia. The pseudopodium makes up between 15-20% of the length of the cell during locomotion. Subpseudopodia may extend from any part of the cell. There is usually no uroid. The granular cytoplasm contains a single nucleus (mean diameter is 4.9 μm) with a single, central nucleolus (mean diameter is 2.4 μm) that is usually more than half the diameter of the nucleus as whole. A large contractile vacuole is located just posterior to the nucleus during locomotion, usually less than one nuclear diameter from the nucleus, and it is usually greater in diameter than the nucleus at diastole. Sporocarps develop as an amoeba rounds up to form a refractile prespore cell that is nearly circular in outline. As the prespore cell develops into a stalk-depositing sporogen, it is more or less spherical. Once stalk deposition is complete, the sporogen develops into a spore either by laying down a spore wall and remaining nearly spherical or, more frequently, changes shape to become ovoid to sausage-shaped to peanut-shaped before laying down a spore wall. Observations have not been made to determine if the spore changes shape continuously as is the case in *L. hula* [14]. The spores are deciduous and flag readily in air currents. The stalks vary considerably in length, but are usually several times longer than the width of the spore. The stalk sits on a basal disk above which is a wide base that accounts for perhaps 5-10% of the total length of the stalk. The stalk then suddenly narrows and the remainder of the stalk is narrow and tapers slightly toward the apex. The very apex of the stalk widens to form a solid-appearing knob-like apophysis that is fully visible when the spore has been shed. The base of the apophysis is visible in the intact sporocarp, suggesting that the apex of the stalk is inserted into a shallow invagination at the base of the spore.

***Vacuolamoeba*** n. g. Tice , Geisen, & Brown 2016

**Diagnosis:** Irregular shaped amoebae, pseudopods variable with anterior hyaloplasmic lamellopodial extensions free of inclusions. Acanthopodial extensions can form from all areas of the cell body. Occasionally cells produce uroid with lamellopodial form that includes filose uroidal extensions. Cells most often with 1 vesicular nucleus with a central nucleolus. Cells have 2 nuclei have been observed. Cell body often has many ca. 4-5 vacuoles, sometimes with one or more contractile vacuoles. Cysts round to irregularly shaped with a single wall. Cysts usually form individually rather than in clusters.

**Type species:** *Vacuolamoeba acanthoformis* n. sp.

***Vacuolamoeba acanthoformis*** n. sp. Tice , Geisen, & Brown 2016

**Diagnosis:** Characteristics of the genus. Mean cell length or breadth is 22.5 μm (standard deviation = 1.4 μm, n = 9). Cells are most often uninucleate with a single round centrally positioned nucleolus. Nucleus diameter ranges from 3.2-5.5 μm (mean = 4.3 μm, standard deviation = 0.8 μm, n = 8). Nucleolus diameter ranges from 1.1-2.2 μm (mean = 1.6 μm, standard deviation = 0.3 μm, n = 8). Mean cyst diameter = 8.0 μm (standard deviation = 1.0 μm, n = 16).

**Type habitat:** High altitude soil from Tibet.

**Type material:** Type culture was deposited at CCAP accession number 2580/1.

**Type sequence:** The partial SSU of the type strain has been deposited on NCBI Genebank accession number KX840328.

**Etymology:** “Vacuol” as this was the first thought upon observation of the large size and prominence of the contractile vacuole(s). “Acantho” latin for “spine” due to the spiny nature of the peudopodia produced and the species initial resemblance to *Acanthamoeba* spp.

**Differential diagnosis:** May upon initial observation resemble both *Acanthamoeba* spp. and *Protacanthamoeba* spp. Spore morphology is the easiest way to distinguish this species from any species of *Acanthamoeba*. Spores of this species are smooth walled and do not exhibit the endocyst/exocyst arrangement typical of most *Acanthamoeba* spp. Differs from *P. bohemica* in that the acanthopoida are not nearly as pronounced.

***Dracoamoeba*** **n.g.** Tice & Brown 2016

**Diagnosis:** amoebae with ramose pseudopodia with the ability to form lamellapodium with acanthapodial subpseudopodia. Pseudopods of all forms made up of hyaloplasm and used for locomotion and feeding. Cell body made of granuloplasm.

**Type species:** *Dracoamoeba jomungandri* n. sp.

***Dracoamoeba jomungandri*** n. sp. Tice & Brown 2016

**Diagnosis:** Characteristics of the genus. When attached to the surface of a culture flask amoebae exhibit long, tapering thin ramose psuedopdia that can for from all sides of the main cell body. Amoebae in this state range from 33 μm - 87 μm (mean = 57.6 μm, standard deviation = 15.6 μm, n = 34) long. The width of the cell body ranges from 3 μm- 12 μm (mean = 6.2 μm, standard deviation = 2.4 μm, n = 34). Psuedopodia are composed of hyaloplasm while the main body of the cell is granuloplasmic in nature. Amoebae do not form uroids. No cysts have been observed. Upon starvation amoebae will shrivel up and detach from the surface. These amoebae will remain suspended in the water column or float on the surface of the water.

**Type habitat:** moist soil from mud flat approximately 800 yards from the ocean, Chincoteague, VA.

**Type material:** type culture deposited with the American Type Culture Collection as *Stereomyxa ramosa*. Accession number 50982.

**Type sequences:** Raw sequence data can be obtained through the MMETSP webportal. Transcriptome accession id MMETSP0439.

**Etymology:** *Dracoamoeba* “Draco” latin meaning “dragon”, as any forms of this amoeba resemble a dragon. “jomungandri” after Jörmungandr, the oceanic sea serpent of norse mythology.

**●**Discosea Cavalier-Smith et al. 2004

●●Centramoebia Cavalier-Smith et al. 2016

●●●Himatismenida Page 1987

(*Cochliopodium, Parvamoeba, Ovalopodium*)

●●●Centramoebida Rogerson & Patterson 2002

●●●● Pellitidae Smirnov and Kudryavtsev 2005

(*Pellita*)

●●●● Goceviidae Smirnov et al. 2011

(*Gocevia*, *Paragocevia*, *Endostelium*)

●●●●Balamuthiidae Cavalier-Smith et al. 2004

(*Balamuthia*)

●●●●Acanthamoebidae Sawyer and Griffin 1975 renewed definition Tice et al. 2016

(*Acanthamoeba, Luapeleamoeba, Protacanthamoeba, Dracoamoeba*, *Vacuolamoeba*)

### Diagnosis

Flattened to dome shaped amoebae with flabellate-type psuedopodia some with furcate subpseudopodia. Where examined a interphase cells with a cytoplasmic microtubular organizing center (MTOC) from laminate structure to a simple globular mass with many raditating microtubules.

**●** *Incertae sedis* Amoebozoa: Stereomyxidae (*Stereomyxa*, *Corallomyxa*)

## Reveiwers’ comments

**Comments and responses to the original submission**

### Reviewer's report 1: Eugene Koonin, National Institutes of Health, USA

Endorse Publication

Reviewer summary

-The manuscript by Tice et al on "Expansion of the Acanthamoebidae (Centramoebida, Amoebozoa)" reports a new phylogeny that adds a variety of diverse protists to this important group. major corollaries of the findings are that the life cycle of some Acanthamoeba includes spore-forming stages and that the overall dominant trend in the evolution of Acanthamoeba involves loss of complexity. The phylogenetic analysis in the paper is state of the art and of high quality.

Reviewer recommendations to authors

-I have no major criticisms. I may note that the title of the paper is somewhat drab. It might be better to indicate that this is a major expansion, and expansion that reveals major biological trends or some such, to more immediately draw attention to the paper.

Also, with regard to the evolutionary trend from complex ancestors to simplified descendants, it might be useful to note the higher generality of this pattern, far beyond Amoebozoa: Wolf YI, Koonin EV. Genome reduction as the dominant mode of evolution. Bioessays. 2013 Sep;35(9):829-37

Author's response: *We agree and have edited the last sentence of the introduction to emphasize this point. " This evolutionary trend of derived simplicity both morphologically and genomically is not only seen in Amoebozoa, but across the tree of Life as a whole* [25, 26]*."*

### Reviewer's report 2: Sandra Baldauf, Uppsala University, Sweden

Endorse Publication

Reviewer Summary:

-Amoebozoa is a generally poorly understood and very unevenly sampled eukaryote supergroup. This includes the major division Acanthamoebidae, despite the fact that it includes important model organisms. The ms reports substantial interesting new data. The most interesting aspect is the origin of sporocarpy, important for understanding basic properties of especially soil microbes, such as dispersal and dormancy. Therefore, the work potentially appropriate for a scientifically broad journal, such as BiolDirect. However, this manuscript lacks coherence and seems to be 2-3 different manuscripts - classical protist taxonomy (Fig. 1), deep phylogeny of Amoebozoa (Fig. 2) and molecular phylogeny of Acanthamoebidae (Additional file 5: Figure S3 and Additional file 6: Figure S2). It was particularly unclear to me what the point of Fig. 2 is. There are also some problems with presentation, but most of these could be easily fixed. I think the manuscript is important and interesting but needs some major revision. You might also consider moving Fig. 2 to a separate manuscript, or if it is included, some additional analyses are recommended and more information on its relevance (detailed below).

Reviewer recommendations to authors:

In general, the manuscript is somewhat lacking in coherence and seems almost like 2-3 different manuscripts - classical protist taxonomy (Fig. 1), deep phylogeny of Amoebozoa (Fig. 2) and molecular phylogeny of Acanthamoebidae (Additional file 6: Figure S3 and Additional file 7: Figure S2).

Author's response: *We have made every effort to clearly unify these three elements (which we feel are all equally necessary) into a single coherent and concise story.*

It was particularly unclear to me what the point of Fig. 2 is.

Author's response: *The Acanthamoebidae and Centramoebida have typically never had high statistical support in molecular phylogenetic reconstructions of Amoebozoa. However, due to the morphological and ultrastructural similarities of the genera that make up the classical composition of the group (i.e. Acanthamoeba and Protacanthamoeba), the validity has never been called into question. We have seen from previous work of ours on Luapeleamoeba hula that due to the drastically different morphology and life cycle of this organism from that of traditional acanthamoebids, reviewers have been highly skeptical of results using SSU alone placing it in Acanthamoebidae. Since the morphologies of both Luapeleamoeba arachisporum and Dracoamoeba jomungandri (deposited as Stereomyxa ramosa ATCC® 50982™ ), and the life cycles of Luapeleamoeba arachisporum and Acanthamoeba pyriformis are equally/more divergent from traditional acanthamoebids than that of Luapeleamoeba hula we chose to use phylogenomics as an additional and possibly more convincing line of evidence for the inclusion of these taxa in the group. Another initial incentive of ours to include this component in our study was the only data available for Dracoamoeba jomungandri (“Stereomyxa ramosa” ATCC® 50982™) was a transcriptome generated by the Marine Microbial Eukaryote Transcriptome Sequencing Project. We wanted to include it in our analyses on Acanthamoebidae since previous phylogenomic studies show it was sister to Acanthamoeba castellanii, but could say no more about the exact phylogenetic placement of this organism due to limited taxon sampling. Despite being able to bioinfomatically find the SSU of this organism in the available transcriptome and thus include it in our SSU analysis, we still feel the above concerns from others in our community, and the traditional lack of support from either ML or Bayesian analyses for the group justify/require the phylogenomic analysis to be included here.*

Specific Points Fig. 1 (taxonomy) The discussion of this figure is >50% of Results, but for a general reader the terminology is inaccessible (furcate, hyaloplasm, lamellopodia, pellitids, etc.) and the detail is of limited utility. About half of this is also confirmation of previous descriptions of the same species. This is all unlikely to be useful for other than a specialist and needs some revision to be more widely accessible (e.g., define terms, move less relevant details to SupDat).

Author's response: *Agreed. The morphological details that would allow experts to feel confident in our identification of previously described species isolated from nature due to their unavailability from any culture collection (i.e. Acanthamoeba pyriformis and Luapeleamoeba arachisporum) have been moved to the supplementary results section. We have also gone through and either replaced specialist terms with more widely understood synonyms or defined them upon their initial use.*

Figure 2 (global rooted phylogeny of Amoebozoa) Most of the Results for this figure focus on the root, which differs from previous work. However, you don't explain why this is relevant here. Perhaps it is meant to show monophyly of Acanthamoebidae & Centramoebids, but you've not made it clear that this is in question.

Author's response: *The purpose of the figure was to show the monophyly of Acanthamoebidae & centramoebids when including organisms we now show to be acanthamoebids, but differ greatly with respects to their morphology and life cycles in some cases from that of traditional acanthamoebids (Acanthamoeba spp. and Protacanthamoeba spp.). In most phylogenetic reconstructions of Amoebozoa using the SSU gene where L. hula is included, neither Centramoebida nor Acanthamoebidae are strongly supported (posterior probability ≥ .95 and ML bootstrap ≥ 80 in our opinion) in both ML and Bayesian analyses (i.e. Shadwick et al. 2009, Lahr et al. 2011, and Berney et al. 2015). In order to clarify this was indeed the purpose of this figure, we have tried to introduce this topic more clearly in the abstract and background sections. Also any discussion about the overall topology or root of the tree has been removed from the results section.*

Also, please explain why other deeply sequenced in-group taxa are not included (e.g. Stereomyxa?)

Author's response: *The transcriptome of “Stereomyxa ramosa” ATCC® 50982™ generated by the Marine Microbial Eukaryote Transcriptome Sequencing Project is included in our analysis as mentioned above. We renamed this strain Dracoamoeba jormungandri, which is a major emphasis of this manuscript, as our light microscope observations on this organism were dramatically different than those of Grell in his original description of S. ramosa. We chose to discuss these inconsistencies between our observations on this organism and those of Grell 1966 in greater detail in a supplementary discussion section as these findings are likely only of interest to specialists. To help with confusion related to old and proposed new names we have also added a table at the suggestion of Reviewer 3 (Purificacion Lopez-Garcia) that includes the names of all isolates used in this study and our suggested new names based on our phylogenetic analyses if relevant. We refer to this table early on.*

Alternatively, if the question is whether more Acanthamoebid data affect deep resolution in Amoebozoa (which seems unlikely), then it would make more sense if this was the last figure. In that case, I also strongly recommend additional controls, such as alternative hypothesis (e.g. AU) tests, and analyses with different outgroups, particularly given the very short internal branches and extremely long terminal ones.

Author's response: *This is not the case. See above.*

Some additional information also - e.g., clearly state that only 2 of 6 PhyloBayes runs converged.

Author's response: *This section has been changed to read:*

*After 1200 generations convergence was achieved for* ***two of the six chains*** *.* ***These two chains*** *were summarized……*

How were problematic sequences vetted (i.e. for what)?

Author's response: *We are not sure how to further clarify this passage from our methods:*

*“To test for undetected paralogy or contaminants, we constructed a consensus tree (ConTree) representing phylogenetic groupings of well-established eukaryotic clades* [35]*. The resulting individual protein trees that placed taxa in conflicting positions relative to the ConTree with more than 70% ML bootstrap support, with a zero-branch length, or with extremely long branches were checked manually. All problematic sequences identified using these methods were removed from the dataset.”*

*Except maybe to say that the sequences that fit the criterion above were deleted from our dataset due to the suspicion that they might represent paralogs or contamination from another organism. These sequences, for obvious reasons, would result in an erroneous phylogenetic signal for a particular organism.*

The methods all use complex models, which come at the expense of adequate search algorithms and rigorous statistical tests. However, the latter are especially important for complex trees (many taxa, widely different rates). If you want to make a strong case for an alternative root, a more comprehensive bootstrap analysis as well as AU tests would be good.

Author's response: *Again, the intentions of the phylogenomic analyses in the manuscript were not to evaluate where the root of Amoebozoa may lie. Our goal was merely to add an additional layer of support for the monophyly of the Acanthamoebidae clade of Centramoebida that includes our morphologically diverse organisms of interest.*

Figure 3 (SSU phylogeny) Most of this section of Results focuses on Additional file 6: Figure S2. This is a nice figure, informative and well-presented. The purpose of using Fig. 3 instead, which does not include all the taxa in question, is not clear. You also don't even mention this figure until half way through this section of Results, which I found confusing.

Author's response: *We agree, and have now moved an aesthetically modified version of Fig. S2 (Now Fig.* *3* *) into the main text. However, we choose to maintain our centramoebid enriched tree (formally Fig.* *3* *now Fig.* *4* *) to show a more precise and more well resolved phylogeny of our group of interest.*

Results The description of trees in Results is mostly a repetition of the names in the figures. This is hard work to read and didn’t add much to my understanding of the main points of the figure. It is _very_ frustrating that taxon labels in the trees are different from those in the text (e.g. Protostelium pyriformis is referred to as such throughout the text but labelled as Acanthamoeba pyriformis in all the trees). This is only explained in the last paragraph of Results. Some higher level taxon names used in the text are also not defined or labeled in the figures (e.g Gocevidae - no indication it includes Endostelium).

Author's response: *We have revised both results sections that discuss tree topologies to take these constructive comments into consideration. We have edited the manuscript in a way that establishes new names of taxa early on and consistently refer to our new names which are displayed on all trees throughout.*

Discussion There are some very interesting points and additional informal observations. However, this is quite long and tends to ramble in places. I think this would be easier to read and have much more impact if you tightened it up a bit.

Author's response: *We have made every attempt to streamline the discussion to focus on the main points.*

In some places there are also multiple layers of speculation, which you should probably keep to a minimum. Homology of sporocarps is critically important, but this is simply stated as a fact. It would greatly help to have documented evidence from micrographs and maybe also diagrams.

Author's response: *We certainly did not intend to word our discussion in a way that would lead readers to assume that homology of sporocarpy across Amoebozoa is a proven fact. This is simply working hypothesis that is a future emphasis of our research endeavors. We have edited this section to make this as clear as possible.*

*When we discuss similarities in sporocarp development or morphology that may indicate potential homology of acanthameobid/centramoebid sporocarpy, numerous citations where these observations were originally documented/discussed or followed up on. As this work has already been done, the need to include additional light micrographs or diagrams of these observations in this particular study.*

I don't think it’s wise to dismiss this under the assumption that genome sequences will solve it. A genome sequence is still a long way from identifying genes responsible for specific traits, particularly for an erratically expressed one. So I expect that this is going to have to rely on ultrastructural evidence for some time.

Author's response: *We agree fully and have edited all such statements to suggest a more holistic approach to tackle this question. This includes techniques that are old and some that very new. We absolutely agree and understand that genomes and transcriptomes are useful and informative tools, but as you point out, especially in non-model organisms we are a long way away from being able to pinpoint exact molecular machinery responsible for particular traits. Although the generation of that type of data is a logical step towards an answer to the intriguing question of sporocarp homology and ancestral amoebozoan complexity.*

I would suggest some caution in putting too much emphasis on the fact that sporocarps are unknown outside Amoebozoa, since you show they are often missed when present, even in Amoebozoa.

Author's response: *We fully understand this recommendation and have edited our introduction of this concept to be more cautious. However, with the results of this body of work considered, of the 33 described species of amoebae known to exhibit protosteloid sporocarpic fruiting the phylogenetic home of only one (Microglomus paxillus) is truly a mystery. All protosteloid amoebae that have molecular data available have found a phylogenetic home in Amoebozoa. The few (aside from M. paxillus) which have none are clearly close relatives to one or more that have been placed with high statistical support within Amoebozoa by molecular phylogenies. Also, all myxogastids sequenced so far form a monophyletic group in Amoebozoa. Many of us who have worked with protosteloid amoebae have also worked with amoebae from a variety of locations across the tree of life. We have grown these amoebae, if possible, in conditions that would facilitate sporocarpic fruiting and have seen none. At least for now we see no reason to believe that sporocarpy has evolved outside of Amoebozoa; however, we do not deny this possibility. The original avenues for some of us, FWS, MWB, LLS, into an interest in amoebozoans was from our surveys for protosteloid amoebae. We have searched the world thoroughly using techniques designed to maximize our chances of finding protosteloid organisms on the basis of their fruiting. We have looked both at traditional terrestrial substrates and submerged freshwater substrates. When they are plated out in a manner to yield sporocarps we have never found any taxa that appear to be other than amoebozoans. Certainly, should it ever come to pass that a sporocarpic non-amoebozoan is discovered, we will happily revise our thinking, but our vast experience suggests that such a finding would have been expected by now.*

Suggestions:

Taxonomy (Fig. 1) - move detailed confirmatory descriptions to SupDat - for the remainder, simplify and define terminology or summarize only and put details in SupDat

Author's response: *Done. As stated above the confirmatory descriptions are now in supplementary discussion. We have also gone through and defined any specialist terms upon their initial use.*

- establish new names early on and use consistently (since these aren’t formal descriptions you could even add them to section headings).

Author's response: *Done. Section headings now include purposed new names for all taxa of interest. Names have also been changed throughout the entirety of text (with the exception of the “Abstract” and “Background” sections) to reflect what is used in the figures. We also hope the previously mentioned table that has been added will help with this.*

Deep phylogeny (Fig. 2) - simplify or delete - if retained - include all Acanthamoebidae/Centramoebidae with substantial genomic data - simplify rest of tree (collapse nodes, delete irrelevant problematic taxa (e.g. archamoebae, probably also outgroup) - or reduce to Discosea and apply root from separate analyses (place latter in SupDat) - clarify what the purpose of these analyses are, and, if the root is the main concern, include additional controls and full bootstrap analyses.

Author's response: *As previously mentioned all deeply sequenced Acanthamoebidae/Centramoebidae lineages were included in our analyses. Again, trying to determine the location of the root of Amoebozoa was not the goal of these analyses. We have made all possible efforts to clarify our intentions in the text. We choose to not remove the figure or alter it beyond the addition of a label for “Centramoebida”, but have made a great effort to clarify early on what the true intention of these analyses were (show that Acanthamoebidae includes are taxa of interest and is fully supported in both ML and Bayesian analyses).*

SSU tree (Fig. 3) - replace Fig. 3 with Fig. 2S, and collapse a few of the more heavily sampled outgroup clades (e.g. most P. mycophaga) (retain full version of 2S in SupDat) Use consistent names - rename OTUs early in Results (can refer forward to SSU tree, if needed) - make sure all higher taxon names used in text are also labelled in the figures (if possible)

Author's response: *As stated above. An aesthetically modified version of Fig. S2 is now included in the main text as Fig.* *3* *. The full version of this tree is retained in supplement as Fig. S2. However, we choose to retain our centramoebid enriched phylogeny (formally Fig.* *3* *now Fig.* *4* *) for reasons mentioned above. All higher taxon names discussed in the text are now labelled on appropriate tree figures.*

Tighten the Discussion - more information on sporocarp morphology in different taxa, including illustrations.

Author's response: *See above.*

-Style - suggest you avoid excessive use of first person and most narrative writing - best to minimise use of hyperbole (greatly increase, deeply affect, cutting-edge…, ) –

Author's response: *All instances have been removed.*

avoid incendiary language (Li. 416 “…based on a profound ignorance of the literature”) –

Author's response: *This statement has been changed.*

avoid referring to hypotheses as “not yet proven” (as opposed to “not yet tested”)

Author's response: *All instances have been removed or changed.*

### Reviewer's report 3: Purificacion Lopez-Garcia, Centre National de la Recherche Scientifique, France

Endorse publication

-In this manuscript, Tice and co-workers contribute to clarifying the phylogeny of Acanthamoebida through phylogenomic analysis and better circumscribing the morphological and cell biology features of the group. Thus, by showing that some amoeba species of previous uncertain position belong to this clade, the range of phenotypic characteristics for this important group of free-living and parasitic amoeba is expanded. While the analyses are well done and the information obtained in this way is relevant for protistologists and other biologists interested in amoeba, the manuscript needs to be significantly improved, as follows.

Major comments: 1. The work is very descriptive and, in many points, excessively specific for the generalist audience of Biology Direct. Therefore, a considerable didactic effort must be done in order to communicate to the broader readership the key points of the message that the authors are trying to pass: i) what was known about this group (a real introduction of the lineages later discussed in the paper), ii) what is the problem that you are addressing (e.g. clarifying the position of certain strains), iii) what your results solve in this respect and iv) what are the further implications of your findings for general amoebal phylogeny and protistology. This information is hidden in the text and it will be very difficult for a non-specialist reader to get the message. I would invite the authors to revise their text accordingly, and eventually restructure some sections, in particular the discussion, to provide a more cohesive manuscript that highlights the important points.

The conclusion section is trivial as it is; I would suggest making a true conclusion of this particular work, or removing it.

Author's response: *This paragraph has been removed.*

2. While some parts of the text can be streamlined (condense excessively detailed and/or hypothetical diversions in the results and discussion) to make your message clearer, there is a significant part of the information missing from the main text. For instance, what is the rational to remove part of materials and methods from the main text while keeping the "325 gene analysis" section? I would suggest including a synthesis of all materials and methods used; an extended, more detailed version can be then included as supplementary material.

Author's response: *Originally we felt that the phylogenomic methods were the most non-standard and would have the broadest appeal to the audience of Biology Direct and as such should be included in the main text. Others (culture maintenance, or transcriptome assembly as two examples) would either only interest specialist or are widely enough used and accepted they would be more appropriately placed in supplementary material.*

*However, the main text now includes minimally an overview of all methods used in this study. Detailed descriptions of methods not already fully described in the main text are retained in the supplementary materials and methods section.*

Also, the species diagnoses must be in the main text.

Author's response: *We agree, the taxonomic appendix that was previously in supplementary material has now been moved into the main text.*

3. Traceability is essential for any work of these characteristics. I suggest you provide a table in the main text including the name of all the Acanthamoebida strains used, the proposed new name (if applicable), the original source of the strain (the type of environment they were isolated from, who isolated them-reference if available) and the accession number in a culture collection. You might also include a few key morphological descriptors. Such a table could greatly improve the presentation of the basic data.

Author's response: *We have added a table (now Table* *1* *) in the main text with the relevant information. We did not include any morphological descriptors however.*

Descriptions of new species and re-descriptions of old ones must be improved at specialist level (include morphometric data, for instance, and full authorship of revised taxa), but these extended descriptions of each species can be included as supplementary information.

Author's response: *These have been added as per request.*

Minor issues

Line 36, abstract, "we greatly increase the diversity". You include only a few additional species to the known diversity, you might perhaps refer to the expansion of morphological traits for the group

Author's response: *This sentence now reads:*

*“Here we expand the known range of morphological and life cycle diversity found in the Acanthamoebidae. . . .”*

Line 42, please define what you mean by 'spore' and 'cyst' for the reader and keep a homogenous nomenclature along the manuscript

Author's response: *Spore and cyst as used here are now defined in the background section.*

Line 51, one key word is "Acanthamoebic keratitis" – this is not mentioned at all in the manuscript.

Author's response: *Removed.*

cited literature, especially in the introduction, is not always relevant and sometimes looks a bit randomly selected. Please, check this.

Author's response: *The introduction has been reworked significantly based on the below recommendation to provide a more thorough introduction to the phylogenetic history of the Acanthamoebidae and the organisms that are the focus of this study. We have done our best to choose the most relevant literature to cite.*

Lines 71-87. This can be summarized in 1-2 sentences, as it is not the topic of this manuscript. By contrast, a more careful presentation of the phylogenetic context (e.g. *Pellita*, *Endostelium* or *Stereomyxa*, which are relevant for your manuscript) would be welcome.

Author's response: *We have revised the introduction in a way that focuses more on the limited range of phenotypes and life cycles found in the group prior to our study and on the phylogenetic history of the group. As a consequence, the section in question has been reduced to one sentence.*

Line 158, "which should be examined…", not a result.

Author's response: *Deleted. This entire section was also moved to supplementary per the suggestion of Sandra Baldauf (Reviewer 2).*

Line 198, define 'uroid.

Author's response: *Done. Sentence now reads: “Uroids (distinct arrangements of cellular extensions at the posterior end of some amoeba species) have been observed….”*

Lines 236-242. This could be removed or shortened, as the taxonomic sampling in your manuscript is not as extensive as that in the mentioned work, so the comparison is limited

Author's response: *This has been removed.*

Line 269. You should include the family labels in the corresponding tree.

Author's response: *Done. All “family” level names have been added to the tree.*

You might consider making a larger (or 2) figure with more or larger pictures where details (e.g. of cysts) are visible

Author's response: *All cyst and spore micrographs have been increased in size within our plate. The micrograph of the P. bohemica cyst has been replaced with a larger one of better quality.*

Additional file 7: Figure S1 does not display any bootstrap value (contrary to what is mentioned in the figure legend).

Author's response: *Corrected. Our figure legend referred to posterior probability values that should have been present at nodes of this tree. They have been added.*

**Comments and responses to the revision**

### Reviewer's report 1: Eugene Koonin, National Institutes of Health, USA

This reviewer made no comments for the revision.

### Reviewer's report 2: Sandra Baldauf, Uppsala University, Sweden

Endorse Publication

Recommendations: The manuscript is now in very good shape. Although it is very taxonomic, I think a single added phrase in the abstract could make it more obvious why the taxonomy is of broader evolutionary importance (see below). The last part of the Discussion is also a bit rough still - I’ve made suggestions below and in the pdf that might help. Also, the last sentence in the Conclusions is a bit garbled. Otherwise, I only have a few minor comments on grammar (below and in the pdf). main points - I suggest a sentence/phrase in the Abstract background pointing out that Acanthamoebidae is not only interesting because of models and pathogens, but also because of sporocarpy, which leads to questions on the evolution of .. (e.g., complexity). - last section of Discussion (Origins of Acanthamoebidae and Centramoebida). This is really interesting but needs fewer general statements and more specific information, e.g. more details on evidence for homology of sporocarpy in different taxa. Specific suggestions on how this can be further investigated/tested would be nice as well, e.g. what is the next step? The section is also mostly about the evolution of sporocarpy, so perhaps a more appropriate title?

Authors' response: *We agree and have made all changes to the manuscript as suggested in the edited PDF. For the statement of " Specific suggestions on how this can be further investigated/tested would be nice as well, e.g. what is the next step? ", we included these types of discussion points within our original submission, but we were requested to remove them. Thus, we have not added these discussion points back.*

Minor issues: Abstract - li. 46-49, awkward sentence - li. 53-56, break up into 2-3 sentences Additional minor suggested edits on the pdf

Authors' response: *We have made these changes. Thank you for the careful consideration of our manuscript.*

### Reviewer's report 3: Purificacion Lopez-Garcia, Centre National de la Recherche Scientifique, France

Endorse Publication

Reviewer Summary:

The manuscript has been considerably improved and I have no further comments

## Additional files

Additional file 1: Tice_etal.2016.SupplementalText.pdf. Supplementary text that includes: an extended materials and methods section, details of light microscope observations, and a discussion on the justification for taxonomic reassignment of *Stereomyxa ramosa* ATCC® 50982™. (PDF 524 kb)
Additional file 2: Bordor.325.refdat.dat. Reference protein dataset used as queries for homology searching in subject databases. (DAT 154 kb)
Additional file 3: Bordor.325.Cent40.tgz. Phylogenomic alignment files and single gene trees for each gene included in the phylogeomic supermatrix. (TGZ 8396 kb)
Additional file 4: Table S1. Details on gene, site sampling, and data sources per taxon used in our phylogenomic dataset. (XLSX 86 KB)
Additional file 5: Figure S3. Fastest evolving site removal assay. Sites were sorted based on their rates of evolution under the model LG?+?G4 as estimated in Dist_Est and removed from the dataset from highest to lowest rate in a stepwise fashion (3,300 AA sites per step). The bootstrap values estimated in IQ-Tree under the model LG?+?G4?+?F and the bootstrap support for each bipartition of interest was plotted. (PDF 9 kb)
Additional file 6: Figure S2. Maximum likelihood phylogeny of Amoebozoa rooted with Ophisthokonta based on the SSU gene and 1,326 nucleotide positions. The tree was constructed under a GTR?+?G?+?I model of nucleotide substitution. The Centramoebida and Protosteliid clades are highlighted and taxa of interest are in bold. Values at nodes are maximum likelihood bootstrap values. (PDF 327 kb)
Additional file 7: Figure S1. UnconvergedChainsPB.pdf. 325 gene (102,140 AA sites) phylogeny of Amoebozoa rooted with Obazoa. The tree was built using PhyloBayes-MPI v1.5a under the CAT?+?GTR model of protein evolution. This tree is the summation of all unconverged chains of Phylobayes. Values at nodes are posterior probabilities. (PDF 206 kb)

## Abbreviations

ATCC: American Type Culture Collection
CCAP: Culture Collection of Algae and Protozoa
ConTree: Consensus tree
DIC: Differential Interference Contrast
ds-cDNA: Double stranded complimentary DNA
ML: Maximum likelihood
MLBS: Maximum likelihood bootstraps
MTOC: microtubular organizing center
PCR: Polymerase Chain Reaction
SSU: 18S small subunit ribosomal RNA

## References

[1] Adl SM, Simpson AG, Lane CE, Lukes J, Bass D, Bowser SS, Brown MW, Burki F, Dunthorn M, Hampl V (2012). The revised classification of eukaryotes. J Eukaryot Microbiol.

[2] Tekle YI, Anderson OR, Katz LA, Maurer-Alcala XX, Romero MA, Molestina R (2016). Phylogenomics of 'Discosea': A new molecular phylogenetic perspective on Amoebozoa with flat body forms. Mol Phylogenet Evol.

[3] Bonkowski M, Brandt F (2002). Do soil protozoa enhance plant growth by hormonal effects?. Soil Biol Biochem.

[4] Clarke M, Lohan AJ, Liu B, Lagkouvardos I, Roy S, Zafar N, Bertelli C, Schilde C, Kianianmomeni A, Burglin TR (2013). Genome of Acanthamoeba castellanii highlights extensive lateral gene transfer and early evolution of tyrosine kinase signaling. Genome Biol.

[5] Geisen S, Fiore-Donno AM, Walochnik J, Bonkowski M (2014). Acanthamoeba everywhere: high diversity of Acanthamoeba in soils. Parasitol Res.

[6] Visvesvara GS, Moura H, Schuster FL (2007). Pathogenic and opportunistic free-living amoebae: Acanthamoeba spp., Balamuthia mandrillaris, Naegleria fowleri, and Sappinia diploidea. FEMS Immunol Med Microbiol.

[7] Siddiqui RaK, NA. Biology and pathogenesis of Acanthamoeba. Parasite Vectors. 2012;5(6). 10.1186/1756-3305-5-6 PMC3284432 22229971

[8] Fiore-Donno AM, Weinert J, Wubet T, Bonkowski M. Metacommunity analysis of amoeboid protists in grassland soils. Sci Rep 2016;6(19068). 10.1038/srep19068 PMC4707496 26750872

[9] Page FC (1981). A Light- and Electron-Microscopical Study ofProtacanthamoeba caledonican. sp., Type-Species ofProtacanthamoeban. g. (Amoebida, Acanthamoebidae)1. J Protozoology.

[10] Smirnov AVG, Goodkov AV (2005). An Illustrated list of basic morphotypes of Gymnamoebia (Rhizopoda, Lobosea). Protistol.

[11] Sawyer TK, Griffin JL. A proposed new family, Acanthamoebidae n. fam. (Order Amoebida), for Certain Cyst-Forming Filose Amoebae. Trans Am Microsc Soc. 1975;94(1):93-8.

[12] Rogerson AP, Patterson DJ. The Naked Ramicristate Amoebae (Gymnamoebae). In: An Illustrated Guide to the Protozoa. Edited by Lee JJL, G.F; Bradbury, P, vol. 2nd ed. Lawrence: Society of Protozoologists; 2002 Dated 2000: pp. 1023–53.

[13] Shadwick LL, Spiegel FW, Shadwick JD, Brown MW, Silberman JD (2009). Eumycetozoa = Amoebozoa?: SSUrDNA phylogeny of protosteloid slime molds and its significance for the amoebozoan supergroup. PLoS One.

[14] Shadwick LLB, Brown MW, Tice, AK, Spiegel, FW. A new amoeba with protosteloid fruiting: Luapeleamoeba hula n. g. n. sp. Acta Protozool. 2016;55(3)123–34.

[15] Page FC (1967). Re-Definition of the Genus Acanthamoeba with Descriptions of Three Species. J Protozool.

[16] Olive LS. The mycetozoans: New York: Academic Press; 1975.

[17] Fiore-Donno AM, Nikolaev SI, Nelson M, Pawlowski J, Cavalier-Smith T, Baldauf SL. Deep phylogeny and evolution of slime moulds (Mycetozoa). Protist. 2010;161(1):55–70. 10.1016/j.protis.2009.05.002 19656720

[18] Bennett WE (1986). An Ultrastractural Study of the Trophozoite and Cyst Stages ofProtostelium pyriformisOlive & Stoianovitch, 1969 (Eumycetozoea, Protosteliia)1. J Protozoology.

[19] Spiegel FW, Gecks SC, Feldman J (1994). Revision of the Genus Protostelium (Eumycetozoa) I: The Protostelium mycophaga Group and the P. irregularis Group. J Eukaryot Microbiol.

[20] Dyková I, Veverkova-Fialova M, Fiala I, Dvorakova H (2005). Protacanthamoeba bohemica sp. n; isolated from the liver of tench, Tinca tinca (Linnaeus 1758). Acta Protozool.

[21] Olive LS (1962). The genus Protostelium. Amer J Bot.

[22] Kudryavtsev A, Brown MW, Tice A, Spiegel FW, Pawlowski J, Anderson OR (2014). A Revision of the Order Pellitida Smirnov et al., 2011 (Amoebozoa, Discosea) Based on Ultrastructural and Molecular Evidence, with Description of Endostelium crystalliferum n. sp. Protist.

[23] Olive LS, Bennett WE, Deasey MC (1984). The New Protostelid Genus Endostelium. Mycologia.

[24] Grell KG (1966). Amoben der Familie Stereomyxidae. Arch Protistenk.

[25] O'Malley MA, Wideman JG, Ruiz-Trillo I (2016). Losing Complexity: The Role of Simplification in Macroevolution. Trends Ecol Evol.

[26] Wolf YI, Koonin EV (2013). Genome reduction as the dominant mode of evolution. BioEssays.

[27] Picelli S, Faridani OR, Bjorklund AK, Winberg G, Sagasser S, Sandberg R (2014). Full-length RNA-seq from single cells using Smart-seq2. Nat Protoc.

[28] Bolger AM, Lohse M, Usadel B (2014). Trimmomatic: a flexible trimmer for Illumina sequence data. Bioinformatics.

[29] Haas BJ, Papanicolaou A, Yassour M, Grabherr M, Blood PD, Bowden J, Couger MB, Eccles D, Li B, Lieber M (2013). De novo transcript sequence reconstruction from RNA-seq using the Trinity platform for reference generation and analysis. Nat Protoc.

[30] The Roger Lab [http://rogerlab.biochem.dal.ca/]. Accessed 2 Nov 2015.

[31] Katoh K, Standley DM (2013). MAFFT Multiple Sequence Alignment Software Version 7: Improvements in Performance and Usability. Mol Biol Evol.

[32] Criscuolo A, Gribaldo S (2010). BMGE (Block Mapping and Gathering with Entropy): a new software for selection of phylogenetic informative regions from multiple sequence alignments. BMC Evol Biol.

[33] Stamatakis A. RAxML version 8: a tool for phylogenetic analysis and post-analysis of large phylogenies. Bioinformatics. 2014. 10.1093/bioinformatics/btu033 PMC3998144 24451623

[34] Le SQ, Gascuel O (2008). An Improved General Amino Acid Replacement Matrix. Mol Biol Evol.

[35] Brown MW, Sharpe SC, Silberman JD, Heiss AA, Lang BF, Simpson AGB, Roger AJ (2013). Phylogenomics demonstrates that breviate flagellates are related to opisthokonts and apusomonads. Proc R Soc B Biol Sci.

[36] Lartillot N, Rodrigue N, Stubbs D, Richer J (2013). PhyloBayes MPI: phylogenetic reconstruction with infinite mixtures of profiles in a parallel environment. Syst Biol.

[37] Rodrigue N, Lartillot N. Site-heterogeneous mutation-selection models within the PhyloBayes-MPI package. Bioinform. 2013;btt729. 10.1093/bioinformatics/btt729 PMC3967107 24351710

[38] Nguyen LT, Schmidt HA, von Haeseler A, Minh BQ (2014). IQ-TREE: A Fast and Effective Stochastic Algorithm for Estimating Maximum-Likelihood Phylogenies. Mol Biol Evol.

[39] Panek T, Zadrobilkova E, Walker G, Brown MW, Gentekaki E, Hroudova M, Kang S, Roger AJ, Tice AK, Vlcek C (2016). First multigene analysis of Archamoebae (Amoebozoa: Conosa) robustly reveals its phylogeny and shows that Entamoebidae represents a deep lineage of the group. Mol Phylogenet Evol.

[40] Wang HC, Susko E, Roger AJ (2014). An amino acid substitution-selection model adjusts residue fitness to improve phylogenetic estimation. Mol Biol Evol.

[41] Susko E, Field C, Blouin C, Roger AJ (2003). Estimation of rates-across-sites distributions in phylogenetic substitution models. Syst Biol.

[42] Galtier N, Gouy M, Gautier C (1996). SEAVIEW and PHYLO_WIN: two graphic tools for sequence alignment and molecular phylogeny. Bioinformatics.

[43] Ronquist F, Teslenko M, van der Mark P, Ayres DL, Darling A, Hohna S, Larget B, Liu L, Suchard MA, Huelsenbeck JP (2012). MrBayes 3.2: Efficient Bayesian Phylogenetic Inference and Model Choice Across a Large Model Space. Syst Biol.

[44] Olive LS, Stoianovitch C (1969). Monograph of the genus Protostelium. Am J Bot.

[45] Olive LS, Stoianovitch C (1960). Two new members of the Acrasiales. Bull Torrey Bot Club.

[46] Dyková IK M (2013). Illustrated guide to culture collection of free-living amoebae.

[47] Spiegel FW, Margulis JOC L, Melkonian M, Chapman D (1990). Phylum plasmodial slime molds, Class Protostelida. Handbook of Protoctista.

[48] Spiegel FWS, Spiegel SL, Keller, HW, Moore DL, Cavender JC. Mycetozoans. In*.* Edited by Mueller GM, Bills GF, Foster MS. Burlington. Biodiversity of Fungi, Inventory and Monitoring Methods. MA: Elsevier Academic Press; 2004: pp. 547-76.

[49] A beginner’s guide to identifying the protostelids [http://slimemold.uark.edu/pdfs/Handbook1_3rd.pdf]. Accessed 21 Mar 2016.

[50] Brown MW, Silberman JD, Spiegel FW (2012). A contemporary evaluation of the acrasids (Acrasidae, Heterolobosea, Excavata). Eur J Protistol.

[51] de Haan M (2014). First records of Protosteloid Amoebae (Eumycetozoa) from the Democratic Republic of the Congo. Plant Ecology Evolution.

[52] Spiegel FW, Stephenson SL (2000). Protostelids of Macquarie Island. Mycologia.

[53] Tice AKF, Fry NW, Stephenson SL (2014). First survey for protosteloid amoebae in South Australia. Mycosphere.

[54] Zahn G, Stephenson SL, Spiegel FW (2014). Ecological distribution of protosteloid amoebae in New Zealand. Peer J.

[55] Shadwick LL (2011). Systematics of protosteloid amoebae.

[56] Lahr DJG, Parfrey LW, Mitchell EAD, Katz LA, Lara E (2011). The chastity of amoebae: re-evaluating evidence for sex in amoeboid organisms. Proc R Soc B Biol Sci.

[57] Berney C, Geisen S, Van Wichelen J, Nitsche F, Vanormelingen P, Bonkowski M, Bass D (2015). Expansion of the ‘Reticulosphere’: Diversity of Novel Branching and Network-forming Amoebae Helps to Define Variosea (Amoebozoa). Protist.

[58] Cavalier-Smith T, Chao EE, Lewis R (2016). 187-gene phylogeny of protozoan phylum Amoebozoa reveals a new class (Cutosea) of deep-branching, ultrastructurally unique, enveloped marine Lobosa and clarifies amoeba evolution. Mol Phylogenet Evol.

[59] Spiegel FW (2011). Commentary on the chastity of amoebae: re-evaluating evidence for sex in amoeboid organisms. Proc R Soc B Biol Sci.

