# Supplementary material for: Expansion of the molecular and morphological diversity of Acanthamoebidae (Centramoebida, Amoebozoa) and identification of a novel life cycle type within the group
Source: Biol Direct. 2016 Dec 28;11:69. doi: 10.1186/s13062-016-0171-0 (PMC5192571; doi:10.1186/s13062-016-0171-0)
Supplement: Additional file 1: — Tice_etal.2016.SupplementalText.pdf. Supplementary text that includes: an extended materials and methods section, details of light microscope observations, and a discussion on the justification for taxonomic reassignment of Stereomyxa ramosa ATCC® 50982™. (PDF 524 kb) [file 13062_2016_171_MOESM1_ESM.pdf]

SUPPLEMENTAL MATERIALS AND METHODS

Culturing and maintenance

Luapeleamoeba hula ATCC® PRA198™, "*Stereomyxa ramosa*" ATCC® 50982™, *Pellita catalonica* ATCC® PRA25™, *Endostelium zonatum* ATCC® PRA191™ were purchased from the American Type Culture Collection®. *Acanthamoeba pyriformis* isolate CR15 and four isolates of *Luapeleamoeba arachisporum* (PKB06-4L-1, AMFD, OG15, CR15) were isolated using techniques outlined for "protostelids" in Spiegel et al. 2007. *Protacanthamoeba bohemica* isolate TT3H was provided by Dr. Martin Kostka. Soil isolates Tib 84 and Tib 168 were isolated and maintained as in Geisen et al. 2014. *Endostelium zonatum*, *Luapeleamoeba hula*, *Acanthamoeba pyriformis,* and all isolates of *Luapeleamoeba arachisporum* were maintained on wMY agar plates (0.002 g malt extract, 0.002 g yeast extract, 0.75 g K_2HPO_4 and 15 g agar / L distilled ddH_2O) inoculated with *Sphingomonas* sp. Strain FLAVO ATCC® BAA-1467™. "*Stereomyxa ramosa*" ATCC® 50982™ was grown in ATCC® medium 1525 (Seawater ATCC® medium 802) in association with *Klebsiella pneumoniae*. *Protacanthamoeba bohemica* was grown on wMY agar plates with *K. pneumoniae* as a food source.

DNA extraction

DNA for PCR from *L. arachisporum* isolate PKB06-4L-1 was obtained using the Chelex method [1] as described in [2]. DNA was extracted from *L. arachisporum* isolate CR15 and *A. pyriformis* isolate CR15 by collecting cells using a flame sterilized inoculation loop and placing them into PCR strip tubes containing 100 µL of QuickExtract (Epicentre, Madison, WI, USA). Tubes were then placed in a thermocycler at 65 °C for 6 min followed by 98 °C for 2 min per the manufacturer's protocol. DNA was extracted from isolate *L. arachisporum* isolate AMFD by flooding and scraping the agar plate containing actively growing amoebae with guanidine buffer (Chomczynski and Sacchi 1987), and transferred to a 1.5 ml centrifuge tube. The pellet obtained after centrifugation was resuspended in 50 µL of guanidine buffer, left at 72 °C for 10 min, centrifuged. The DNA in the supernatant was precipitated with 50 µL of isopropanol and kept overnight at -20 °C. After centrifugation, the pellet was resuspended in 30 µl of buffer AE of the DNeasy plant mini-kit (Qiagen, Hilden, Germany). DNA extraction for isolates Tib 84 and Tib 168 were performed as described in [3].

PCR of SSU rRNA genes

The SSU rDNA gene sequences were obtained for *L. arachisporum* isolates PKB06-4L-1, CR15, and *A. pyriformis* isolate CR15 by PCR using the universal eukaryotic primers "A" and "B" [4]. Reactions were done in 25 μL total volume using GoTaq® Green Master Mix (promega, Madison, WI). The cycling parameters for *L. arachisporum* isolate PKB06-4L-1 are the same implemented in [5]. The following conditions were used for *L. arachisporum* isolate Pa-CR15 and *A. pyriformis* isolate CR15: 95 °C for 3 min, followed by 35 cycles of 95 °C 30 s, 50 °C for 25 s, and 72 °C for 2.5 min ending with a single cycle of 72 °C for 5 min. The nearly complete SSU sequence of *L. arachisporum* AMFD was obtained in two overlapping fragments, with the following combinations of primers: S1/SR13, and S12m/RibB [6]. Amplification parameters for *L. arachisporum* isolate AMFD were as follows: 94 °C, 2 min, followed by 30 cycles of 94 °C, 30 s; 53 °C, 30 s; 72 °C, 1 min; a final elongation step at 72 °C for 5 min. Amplicons of *L. arachisporum* isolates PKB06-4L-1 and Pa-CR15 along with *A. pyriformis* isolate CR15 were ligated into the pCR4-TOPO plasmid (Invitrogen, Carlsbad, CA) and transformed into Top10 chemically competent *Escherichia coli* cells. Recombinant plasmid clones were fully sequenced in both orientations using vector and internal primers by Sanger Sequencing. PCR and sequenceing for isolates Tib 84 and Tib 168 were performed as described in [3].

cDNA library preparation and next-generation sequencing

A loop made from 0.20 mm platinum wire (Surepure Chemetals, Florham Park, NJ, USA) was used to collect ~50-100 cells from a feeding front of *A. pyriformis* isolate CR15 and *P. bohemica* isolate TT3H. The loops containing amoebae were each placed into separate 200μL-microcentrifuge tubes containing a lysis buffer [7]. Total RNA was extracted from both using a modified version of Smart-seq2 [7]. The modification to the protocol was the addition of six rounds of a freeze-thaw cycle in -80 °C isopropanol and ~21 °C H_2O respectively to assure that lysis of the cells occurred. The result from this protocol is full-length double stranded cDNA. This ds-cDNA is further quality controlled as follows; each ds-cDNA preparation is quantified using Qubit High Sensitivity dsDNA Assay (Life Technologies, Carlsbad, CA, USA). Then the Qubit assay (1:200 dilution of the ds-cDNA preparation) was used as a template for a size PCR assay. The PCR reaction includes 1.0 μL (1:200 dilution), 6.25 μL GoTaq Green Master Mix (2x) (Promega, Madison, Wisconsin, USA), 1.0 μL ISPCR (5'- AAG CAG TGG TAT CAA

CGC AGA GT (Picelli et al. 2014)) primer (10 µM) and 4.25µL PCR-grade H_2O. PCR conditions: 98 °C 3 min, 21 cycles (98 °C 20 s, 67 °C 15 s, 72 °C 6 min), 72 °C 5 min, and hold 4 °C. After this PCR run, the reactions were electrophoresed on a 1.8% agarose gel in Tris-Boric Acid EDTA (TBE) with ethidium bromide and imaged under a UV transilluminator.

The resultant ds-cDNA generated from the Smart-seq2 method [7] is used as the starting material for tagmentation with a Nextera XT DNA Library Preparation Kit (Illumina, San Diego, USA) following the manufacturer's protocol. The resultant dual-indexed Illumina libraries were quantified using Qubit High Sensitivity dsDNA Assay. The size of each library was estimated as follows; each library quantification reaction used for the Qubit assay (1:200) was used as a template for subsequent PCR of each Illumina indexed library using Then the Qubit assay (1:200 dilution of the Nextera XT library) was used as a template for a size PCR assay. The PCR reaction includes 1.0 µL (1:200 Qubit assay dilution), 5.0 µL GoTaq Green Master Mix, 0.5 µL IlluminaF (5' – AAT GAT ACG GCG ACC AC) at 10 µM and 0.5 µL IlluminaR (3' – CAA GCA GAA GAC GGC AT) at 10µM (Oligonucleotide sequences © 2016 Illumina, Inc. All rights reserved), and 3.0 µL PCR-grade H_2O. PCR conditions: 98 °C 45 s, 20 cycles (98 °C 25 s, 47 °C 30 s, 72 °C 1.5 min), and hold 4 °C. After this PCR the reactions were run on a 1.8% agarose gel in TBE with ethidium bromide and imaged under a UV transilluminator. The average size of the library is estimated from this 1.8X TBE agarose gel. Library molarities are calculated using the follow approach. First the average molecular weight (MW) of each library is calculated by MW=(Average Library Size in basepairs * 607.4+157.9). The nanomolarity of each library is calculated by nM=(MW/Qubit Concentration (in ng/ul)*1,000,000).

SSU sequences acquired from transcriptomes

Raw sequence data from the RNAseq project of "*Stereomyxa ramosa*" ATCC® 50982™ was obtained from the Marine Microbial Eukaryote Transcriptome Sequencing Project (MMETSP) (MMETSP0439) [8] and assembled in-house in order to search for the SSU rRNA gene within the data. The SSU rRNA gene from both the above "*Stereomyxa ramosa*" ATCC® 50982™ data and our *P. arachiporum* OG15 data were obtained bioinformatically from the transcriptomic assemblies. To do this clusters from the assemblies of "*Stereomyxa ramosa*" and our *L. arachisoporum* OG15 data were collected and a blastable nucleotide dataset was complied from

each. The SSU rRNA gene was identified in both of these taxa using *Acanthamoeba astronyxis* (AF019064) SSU rRNA gene as a blastn query.

SSU rDNA phylogenetics

To test if our isolates *L. arachisporum* and *A. pyriformis* (originally described as *Protostelium arachisporum* and *Protostelium pyriformis* respectively) could have genetic affinities with other *Protostelium* or *Planoprotostelium*, we assemble their SSU sequences with the 35 sequences available in GenBank, to a seed alignment of 77 other representative amoebozoan sequences and 16 opisthokont sequences as an outgroup. In order more precisely to establish the relationships of our isolates to other centramoebids the eight novel SSU sequences obtained here were added to a dataset comprised of 35 centramoebid sequences. We used six members of Himatismendida as a close outgroup (based on results from our multigene analysis), allowing for the useage of more phylogenetically informative sites than the more taxon rich dataset above. Sequences for both data sets were aligned using MAFFT v7 [9] and ambiguous sites were removed by hand in Seaview v3.2 [10]. Maximum likelihood (ML) trees for both data sets were built using RAxML v8.2.4 under a GTR + Γ + I model of nucleotide substitution (25 discrete rate categories). The topology of each best-scoring tree was evaluated by 1,000 ML bootstrap replicates. Bayesian analyses were performed on the Centramoebida + Himatismenida data set using MrBayes v3.2.6 [11]. Two simultaneous MCMC runs of 4 chains each were run for 10,000,000 generations saving trees every 1,000 generations. All parameters converged after the first 326,000 generations as assessed by split deviation of < 0.01. The initial 25% of trees were discarded as burnin.

SUPPLEMENTAL RESULTS

Acanthamoeba pyriformis CR15

The sporocarps of our isolate consist of a single spore atop a long stalk (Fig. 1: A). The spores on stalks wave or “flag” readily when any air current is generated https://youtu.be/HvzflpE-6Fk. Spores at higher magnification are pyriform in shape and appear to have only a single wall (Fig. 1 B & C). In addition to these sporocarps, our isolate also rarely forms irregularly shaped stellate cysts on the surface of the agar (Fig. 1 E). The amoebae that germinate from both spores and

cysts are uninucleate with a single central round nucleolus that is half to less than half the diameter of the whole nucleus (Fig. 1 D & F). The majority of the cytoplasm is granuloplasm, with a clearly demarcated section of hyaloplasm in the anterior part of the cell (Fig. 1: D & F). Thin, pointed subpseudopodia project outwards from nearly the entire body of the cells (Fig. 1 D & F). A single contractile vacuole that is round at diastole is usually present and located near and posterior to the nucleus (Fig. 1 D & F). The amoebae generally lack a uroid. These observations are consistent with what has been previously reported for this species [12, 13].

Luapeleamoeba arachisporum isolates PKB06-4L-1, OG15, CR15, AMFD

The sporocarps and amoebae of all isolates fall within the range of the description given for "*Protostelium*" *arachisporum* by [14]. The sporocarps are unispored and long-stalked (Fig. 1 J). The spores are either spherical to ovate or peanut shaped depending on their state of hydration (Fig. 1 J & K). After germination from the spore the amoebae leave behind a very thin spore wall (Fig. 1 K). The amoebae are uninucleate, and a central, round nucleolus that is half the diameter or greater than the nucleus as a whole is usually visible (Fig. 1 L). Amoebae have flabellulate-type pseudopodia [15] (Fig 1 L & K) and cells lack a distinct uroid. These flabellulate psuedopodia are hyaloplasmic in their composition and have subpseudopodia that are short and blunt (Fig 1 L & K). Cysts were rarely observed in isolates PKB06-4L-1 and CR15 and never seen in any of our other isolates. When cysts did form they resemble rounded amoebae rather than an obviously walled cyst as reported for "*Protostelium*" *arachisporum* [13]. However, when the amoebae germinate from these cysts they leave behind a very thin covering (not shown).

Vacuolamoeba acanthaformis n.g. n.sp.

The majority of the body of the cell consists of granuloplasm (cytoplasm with inclusions such as organelles) while the leading edges of cells in motion are made up of hyaloplasm (clear cytoplasm lacking any inclusions). Acutely pointed subpseudopodia of acanthopodial morphology project outwards from all sides of the cell body. At times they can be particularly prominent at the leading edge of amoebae in motion. Cells are typically uninucleate although binucleate individuals were sometimes observed. The nucleus is vesicular and displays a round centrally located nucleolus that is half or less than half the diameter of the whole nucleus. Cells typically have many vacuoles in the granuloplasm; among them one or more large round

contractile vacuoles is usually present (Fig. 1 P, Q, & S). Anterior and posterior poles of the cell are often difficult to distinquish, and as a consequence the direction of locomotion is difficult to predict https://youtu.be/X617mOS1ac4. However, amoebae sometimes move by a quick swelling of the leading edge of the hyaline area. When the acanthopodia at the leading edge are prominent this swelling action occurs just behind/at the base of the acanthopodia. When acanthopodia at the leading edge are less pronounced the motion is less of a swelling action and more of a smooth and gradual advancement of the hyaloplasm. Cell motion is slow, but easily visible when observed under the microscope. Uroids (distinct arrangements of cellular extensions at the posterior end of some amoeba species) have been observed, but seem to be missing more often than present. When they do form they are lamellipodial with tiny filose extensions around the outside. Cells readily form cysts in culture. These cysts are round to slightly irregular in shape and consist of what appears at the light microscope level to be a single smooth wall enclosing granuloplasm. The cysts were most often seen to form singly rather than in clusters.

Dracoamoeba jomungandri n.g. n.sp. ATCC® 50982™ (depostied as Stereomyxa ramosa)

The amoebae of ATCC® 50982™ are highly variable in their morphology (Fig. 1 T-AA). Amoebae that have been left undisturbed in tissue culture flasks most often appear thin and ramose, and typically display a thick swelling of cytoplasm that we consider the main body of the cell. The amoebae can also be compact and fan shaped (Fig. 1 U), or exhibit forms that are compact with filose extensions protruding out from various areas around the cell body (Fig 1. V, W Y, Z, & AA). The compact and fan-shaped stage (Fig 1. I) seems to be exhibited only when amoebae are reattaching to a surface after having been displaced into the water column. The cell body is composed mainly of granuloplasm while pseudopods are clear and mostly hyaloplasmic in nature. Though occasionally a small number of granular cytoplasmic content could be seen in the basal region of some pseudopods. Only uninucleate individuals were seen. However, nuclei are not always easily visible especially in the most thin and filose forms of this organism so we cannot say definitively that individuals with more than one nucleus are not present. When observed, the nuclei resemble an irregular clear spot in the cell (Fig 1. V, & W, & Y). No nucleoli are obvious using light microscopy. As expected for a marine organism, amoebae were never seen to form contractile vacuoles. Only food vacuoles were observed. No cysts or resting stages were ever seen in our cultures. When cultures are left to starve, rather than encyst the

amoebae shrivel up and float either in the water column or at the surface of the water. Movement of the amoebae is extremely slow and most easily viewed using time-lapse microscopy (https://youtu.be/UyI1rZPOzAM). Uroids were never observed. No anastomosis or any form of fusion of pseudopodia within or between individuals was ever observed. The characteristics of this organism do not adequately fit the original description given by [16] for *Stereomyxa ramosa* and so we establish here the new genus *Dracoamoeba* n. g. to accommodate this organism and designate this isolate the type species *Dracoamoeba jomungandri* n. g. n. sp. A taxonomic diagnosis of this strain is given in the supplemental text. For a full discussion on the inconsistencies between our observations and those of Grell see the supplemental discussion.

SUPPLEMENTAL DISCUSSION

Identity of isolate ATCC® 50982™

While it is not unimaginable that this organism was deposited as *Stereomyxa ramosa* as it is an amoeba that is: typically uninucleate, slow moving, and marine, we are confident that we have made key observations suggesting this was a misidentification. Grell specifically mentions in his 1966 manuscript that upon starvation, the amoebae of *S. ramosa* do not float like those of a second species he describes in the same manuscript, *S. angulosa*, but remain attached to the surface of the culture dish [16]. In all of our cultures that are left to starve, hundreds of shriveled

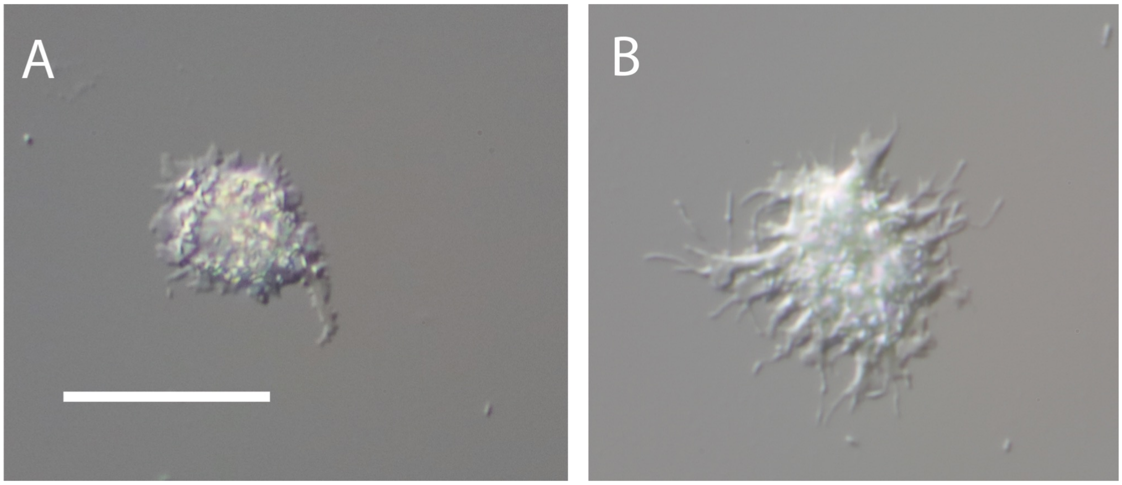

Fig. A, B. Amoebae of *Dracoamoeba jomungandri* n.g. n. sp. ATCC® 50982™ in 3X concentrated seawater.

amoebae can be observed floating in the water column. We do not believe ATCC® 50982™ is *S.*

angulosa either. The time lapse videos made by Grell [17] of *S. angulosa* clearly show an amoeba with radically different morphology and locomotion than we have observed in ATCC® 50982™ (Fig 1. T-AA & https://youtu.be/UyI1rZPOzAM). Grell also states the floating "hunger" form of *S. anulosa* is often times an acute angle. We have never observed this morphology in ATCC® 50982™. Another key observation supporting our claim is that when placed in 3X seawater the Stereomyxidae (*Stereomyxa* spp. and *Corallomyxa mutabilis*) of Grell become "hairy" in appearance and eventually adopt a "beads on a string" morophology. Grell considered this reaction indicative of all Stereomyxidae. When placed in 3X seawater the amoebae of ATCC® 50982™ do become slightly "hairy" in appearance as Grell describes, but no more than we have seen the amoebae appear when attempting to reattach to the substrate after being displaced in the water column (See above. Fig. A & B: Amoebae of *D. jomungandri* in 3X seawater. Scale bar = 25μm). Most importantly the amoebae never assume the "beads on a string" form Grell considered diagnostic of all Stereomyxidae[16]. Also, Grell also used an unidentified species of marine diatom as a food source for both species of *Stereomyxa* in his manuscript. All of our attempts to grow ATCC® 50982™ on diatoms as a food source failed. No diatoms were ever observed in food vacuoles of this species. Masses of empty diatom frustules that might suggest the amoebae were consuming the alga through means other than phagocytosis of whole individuals were never observed either. At present no ultrastructural data are available for ATCC® 50982™ so we are unable to compare these potentially diagnostic details to those of [18]. Despite our lack of ultrastructural data we believe our morphological and experimental observations justify the creation of a new genus for this isolate. We place it into our newly established genus *Dracoamoeba* n. g. n. sp. We designate this isolate as the type species *Dracoamoeba jomungandri* n. g. n. sp. As such we remove all members of Grell's Stereomyxidae from Centramoebida and place them as Amoebozoa incertae sedis until they can be reisolated and studied using modern molecular phylogenetic techniques.

Supplemental Literature Cited

1. Singer-Sam JT, R. C; Riggs A. D: **Use of Chelex to improve the PCR signal from a small number of cells**. *Amplifications* 1989, **3**(11).

2. Shadwick LL, Spiegel FW, Shadwick JD, Brown MW, Silberman JD: **Eumycetozoa = Amoebozoa?: SSUrDNA phylogeny of protosteloid slime molds and its significance for the amoebozoan supergroup**. *PloS one* 2009, **4**(8):e6754.
3. Geisen S, Fiore-Donno AM, Walochnik J, Bonkowski M: **Acanthamoeba everywhere: high diversity of Acanthamoeba in soils**. *Parasitology research* 2014, **113**(9):3151-3158.
4. Medlin L, Elwood HJ, Stickel S, Sogin ML: **The characterization of enzymatically amplified eukaryotic 16S-like rRNA-coding regions**. *Gene* 1988, **71**(2):491-499.
5. Brown MW, Spiegel FW, Silberman JD: **Amoeba at Attention: Phylogenetic Affinity of Sappinia pedata**. *The Journal of eukaryotic microbiology* 2007, **0**(0):071116223551002-???
6. Fiore-Donno AM, Meyer M, Baldauf SL, Pawlowski J: **Evolution of dark-spored Myxomycetes (slime-molds): Molecules versus morphology**. *Molecular phylogenetics and evolution* 2008, **46**(3):878-889.
7. Picelli S, Faridani OR, Bjorklund AK, Winberg G, Sagasser S, Sandberg R: **Full-length RNA-seq from single cells using Smart-seq2**. *Nature protocols* 2014, **9**(1):171-181.
8. Keeling PJ, Burki F, Wilcox HM, Allam B, Allen EE, Amaral-Zettler LA, Armbrust EV, Archibald JM, Bharti AK, Bell CJ *et al*: **The Marine Microbial Eukaryote Transcriptome Sequencing Project (MMETSP): illuminating the functional diversity of eukaryotic life in the oceans through transcriptome sequencing**. *PLoS biology* 2014, **12**(6):e1001889.
9. Katoh K, Standley DM: **MAFFT Multiple Sequence Alignment Software Version 7: Improvements in Performance and Usability**. *Molecular biology and evolution* 2013, **30**(4):772-780.
10. Galtier N, Gouy M, Gautier C: **SEAVIEW and PHYLO_WIN: two graphic tools for sequence alignment and molecular phylogeny**. *Bioinformatics* 1996, **12**(6):543-548.
11. Ronquist F, Teslenko M, van der Mark P, Ayres DL, Darling A, Hohna S, Larget B, Liu L, Suchard MA, Huelsenbeck JP: **MrBayes 3.2: Efficient Bayesian Phylogenetic Inference and Model Choice Across a Large Model Space**. *Systematic biology* 2012, **61**(3):539-542.
12. Bennett WE: **An Ultrastractural Study of the Trophozoite and Cyst Stages ofProtostelium pyriformisOlive & Stoianovitch, 1969 (Eumycetozoea, Protosteliia)1**. *The Journal of Protozoology* 1986, **33**(3):405-411.
13. Olive LSS, C: **Monograph of the genus Protostelium**. *Am J Bot* 1969, **56**(9):979-988.
14. Olive LS: **The genus Protostelium**. *Amer J Bot* 1962, **49**:297-303.
15. Smirnov AVG, A. V.: **An Illustrated list of basic morphotypes of Gymnamoebia (Rhizopoda, Lobosea)**. *Protistology* 2005, **1**:20-29.
16. Grell KG: **Amoben der Familie Stereomyxidae**. *Arch Protistenk* 1966, **109**(19):147-154.
17. **Grell's Stereomyxa** [http://dx.doi.org/10.3203/IWF/C-942 - t=03:36,04:19]
18. Benwitz VG, ; Grell, K. J. : **Ultrastruktur mariner Amoben II. Stereomyxa ramosa**. *Arch Protistenkd* 1971.
