# Supplementary figures and images for: Expansion of the molecular and morphological diversity of Acanthamoebidae (Centramoebida, Amoebozoa) and identification of a novel life cycle type within the group
Source: Biol Direct. 2016 Dec 28;11:69. doi: 10.1186/s13062-016-0171-0 (PMC5192571; doi:10.1186/s13062-016-0171-0)

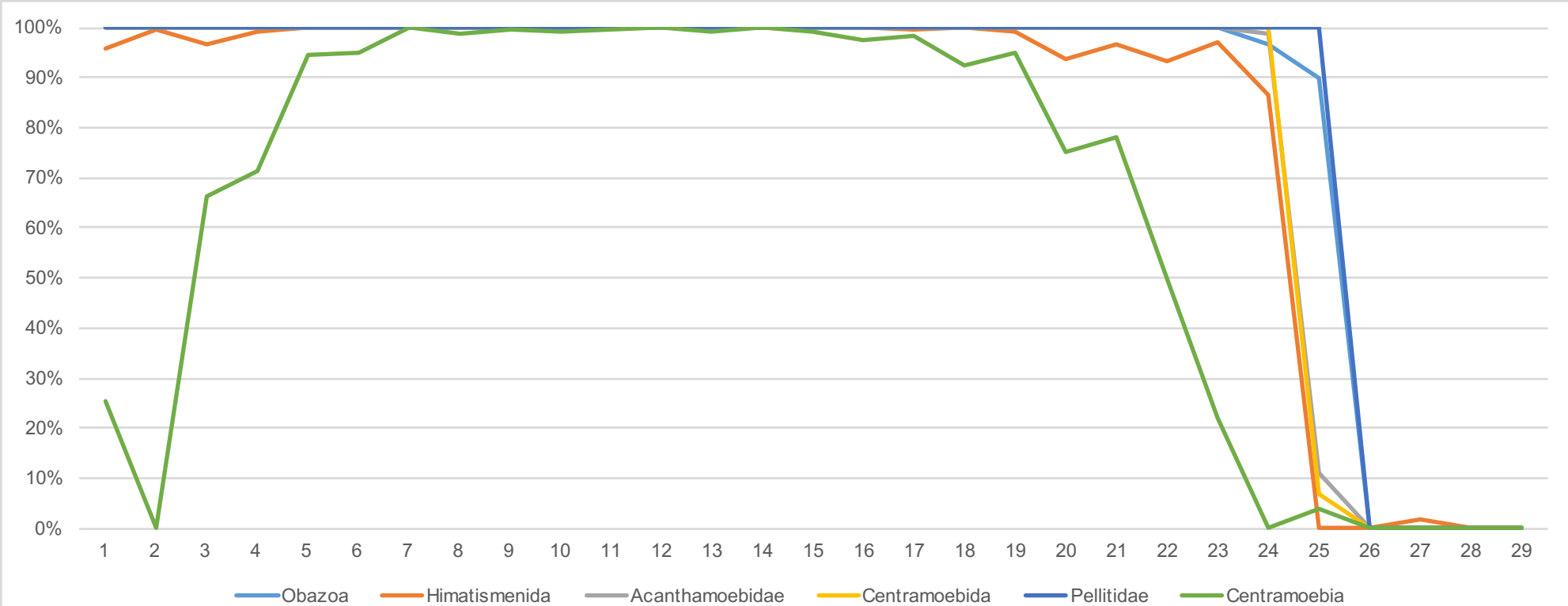

Supplement: Additional file 5: Figure S3. — Fastest evolving site removal assay. Sites were sorted based on their rates of evolution under the model LG?+?G4 as estimated in Dist_Est and removed from the dataset from highest to lowest rate in a stepwise fashion (3,300 AA sites per step). The bootstrap values estimated in IQ-Tree under the model LG?+?G4?+?F and the bootstrap support for each bipartition of interest was plotted. (PDF 9 kb) [file 13062_2016_171_MOESM5_ESM.pdf]

# Centramoebida

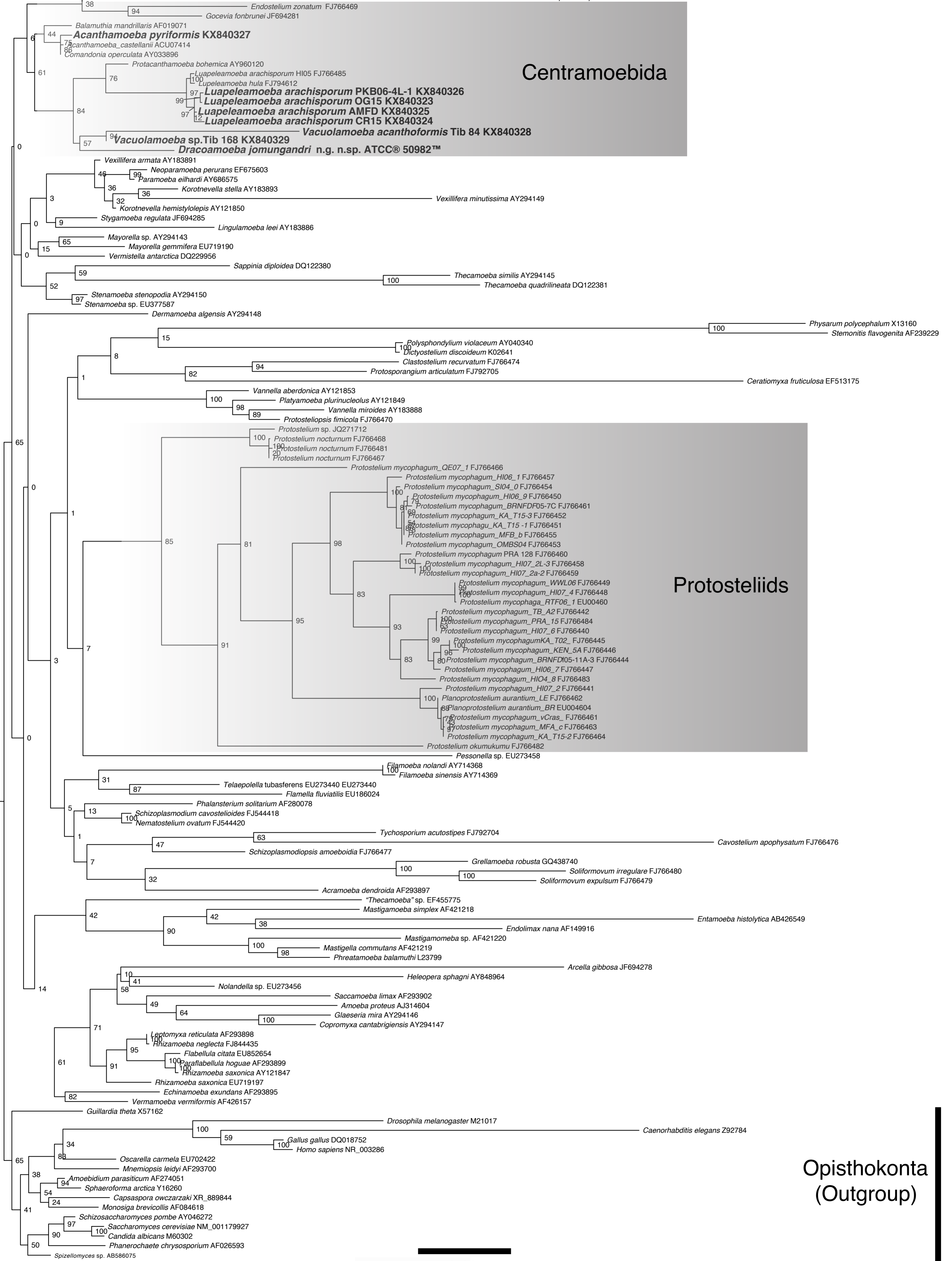

## Protosteliids

## Opisthokonta (Outgroup)

Supplement: Additional file 6: Figure S2. — Maximum likelihood phylogeny of Amoebozoa rooted with Ophisthokonta based on the SSU gene and 1,326 nucleotide positions. The tree was constructed under a GTR?+?G?+?I model of nucleotide substitution. The Centramoebida and Protosteliid clades are highlighted and taxa of interest are in bold. Values at nodes are maximum likelihood bootstrap values. (PDF 327 kb) [file 13062_2016_171_MOESM6_ESM.pdf]

# Acanthamoebidae

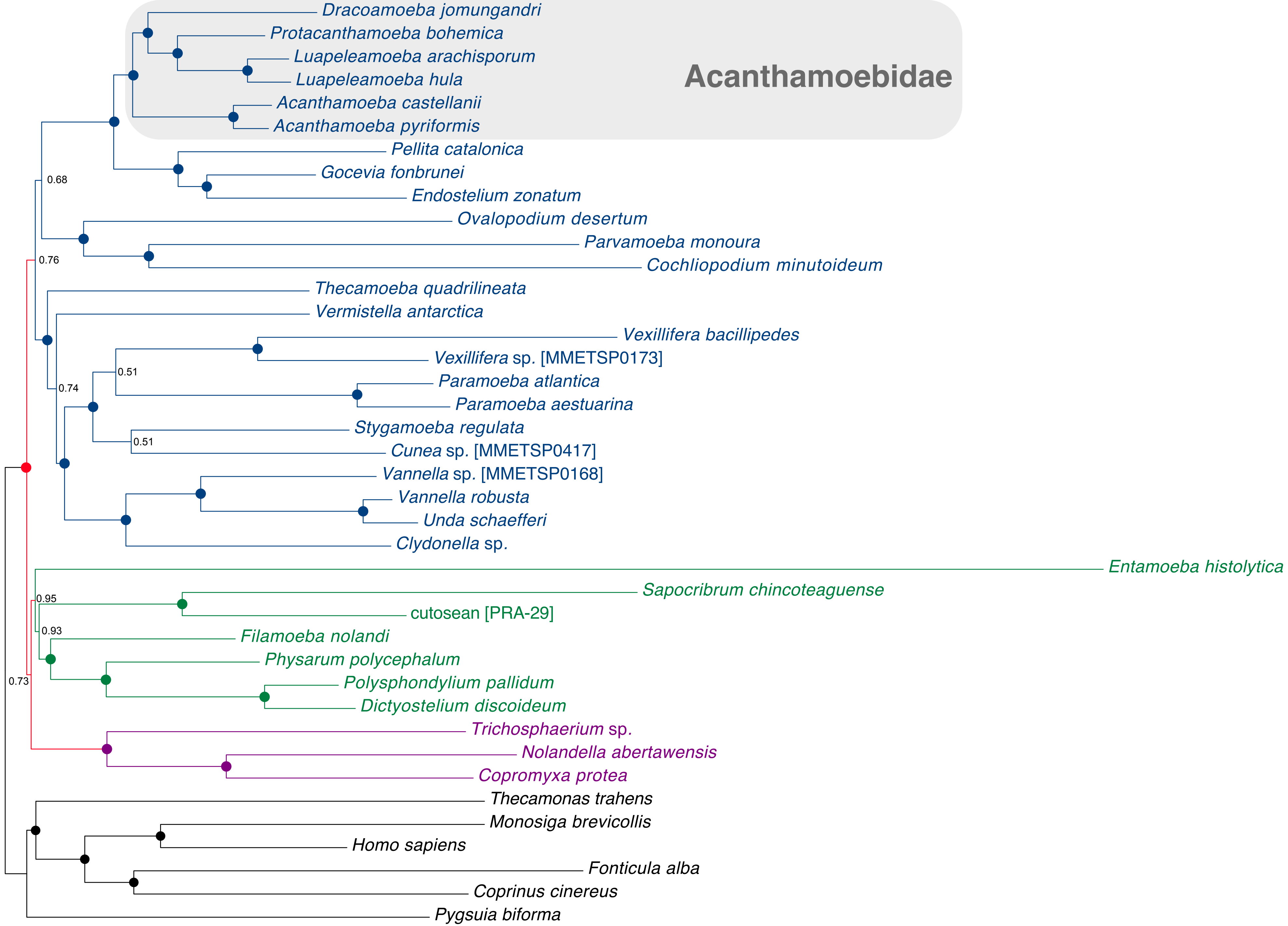

Supplement: Additional file 7: Figure S1. — UnconvergedChainsPB.pdf. 325 gene (102,140 AA sites) phylogeny of Amoebozoa rooted with Obazoa. The tree was built using PhyloBayes-MPI v1.5a under the CAT?+?GTR model of protein evolution. This tree is the summation of all unconverged chains of Phylobayes. Values at nodes are posterior probabilities. (PDF 206 kb) [file 13062_2016_171_MOESM7_ESM.pdf]
